# Crucial role and conservation of the three [2Fe-2S] clusters in the human mitochondrial ribosome

**DOI:** 10.1016/j.jbc.2024.108087

**Published:** 2024-12-13

**Authors:** Linda Boß, Oliver Stehling, Hans-Peter Elsässer, Roland Lill

**Affiliations:** Institut für Zytobiologie im Zentrum für Synthetische Mikrobiologie SynMikro, Philipps-Universität Marburg, Marburg, Germany

**Keywords:** protein synthesis, iron-sulfur protein assembly, biogenesis, mitochondria, mitochondrial respiratory chain, ribosome assembly

## Abstract

Mitochondria synthesize only a small set of their proteins on endogenous mitoribosomes. These particles differ in structure and composition from both their bacterial 70S ancestors and cytosolic 80S ribosomes. Recently published high resolution structures of the human mitoribosome revealed the presence of three [2Fe-2S] clusters in the small and large subunits. Each of these clusters is coordinated in a bridging fashion by cysteine residues from two different mitoribosomal proteins. Here, we investigated the cell biological and biochemical roles of all three iron-sulfur clusters in mitochondrial function and assembly. First, we found a requirement of both early and late factors of the mitochondrial iron-sulfur cluster assembly machinery for protein translation indicating that not only the mitoribosome [2Fe-2S] clusters but also the [4Fe-4S] cluster of the mitoribosome assembly factor METTL17 are required for mitochondrial translation. Second, siRNA-mediated depletion of the cluster-coordinating ribosomal proteins bS18m, mS25, or mL66 and complementation with either the respective WT or cysteine-exchange proteins unveiled the importance of the clusters for assembly, stability, and function of the human mitoribosome. As a consequence, the lack of cluster binding to mitoribosomes impaired the activity of the mitochondrial respiratory chain complexes and led to altered mitochondrial morphology with a loss of cristae membranes. Finally, *in silico* investigation of the phylogenetic distribution of the cluster-coordinating cysteine motifs indicated their presence in most metazoan mitoribosomes, with exception of ray-finned fish. Collectively, our study highlights the functional need of mitochondrial iron-sulfur protein biogenesis for both protein translation and respiratory energy supply in most metazoan mitochondria.

The mitochondrion, an organelle of endosymbiotic origin, is mainly known as the energy-supplying powerhouse of eukaryotic cells. However, it also participates in numerous other critical functions such as fatty acid metabolism, biosynthesis of iron-sulfur (Fe/S) clusters, heme, and lipoate, as well as apoptosis. Like chloroplasts, another endosymbiotic organelle, mitochondria have retained a reduced genome, which in human codes for 13 protein subunits of the respiratory chain, 22 tRNAs, and the 12S and 16S rRNAs (1). For the generation of these proteins and RNAs, the mitochondrion relies on a comparatively simple gene expression apparatus. This includes replication and transcription machinery with DNA and RNA polymerases, as well as translation factors and mitochondrial ribosomes (mitoribosomes) that functionally resemble bacterial counterparts (2, 3). During protein synthesis at the mitochondrial inner membrane, the translation products are cotranslationally inserted into the mitochondrial respiratory complexes, requiring coassembly with numerous subunits imported from the cytosol after translation on cytosolic 80S ribosomes (4).

Recent structural and biochemical research on mitochondrial protein translation unveiled a detailed picture of the mammalian mitoribosome architecture (5, 6, 7, 8) and provided insights into its complex assembly pathways (9, 10, 11, 12, 13) and the various stages of the translational process (14, 15). Apart from the conserved catalytic core and basic functionality of the mitoribosome, the particle has undergone a remarkable structural evolution away from its bacterial origins to specialize for its function in translating few mitochondrial inner membrane proteins, many of which are highly hydrophobic. Compared to the bacterial 70S ribosome, the mammalian 55S mitoribosome possesses an inversed protein: rRNA ratio of 2:1. Additionally, recent studies have revealed a great structural and compositional variety among eukaryotic mitoribosomes (16). For instance, the protozoan mitoribosome shows an unusually high protein: rRNA ratio of 6:1, and a recent structure from the algae *Chlamydomonas reinhardtii* disclosed the presence of 13 rRNA pieces (17).

An unexpected discovery was the presence of two [2Fe-2S] clusters in the 28S small subunit (SSU) and one [2Fe-2S] cluster in the 39S large subunit (LSU) of the human mitoribosome (6). Initially, the three Fe/S cluster-binding sites were annotated as Zn ion-containing sites, and only high-resolution structures could unequivocally resolve the three [2Fe-2S] clusters. Moreover, three Zn ions were found in the human mitoribosome, each coordinated by a single protein subunit (mS40, bL32m, and bL36m) *via* four cysteine (Cys) residues. The three human mitoribosome [2Fe-2S] clusters are coordinated in a unique fashion by two mitochondrial ribosomal proteins (MRPs) for each cluster, one MRP providing three and the other one Cys residue for ligation. The three MRP subunit pairs are mL66-uL10m, mS25-bS16m, and bS18m-bS6m. Intriguingly, mL66, bS18m, and Zn-binding mS40 share high sequence and structural similarity, with mL66 and mS40 possibly having originated from gene duplication (8). Currently, only two additional species have been experimentally shown to contain Fe/S clusters within their ribosomes, namely the bacterium *Thermus thermophilus* (18) that binds a [4Fe-4S] cluster *via* the ribosomal SSU protein S4, and *C. reinhardtii* that coordinates a [2Fe-2S] cluster in its mitochondrial LSU by the algae-specific mL119 protein (17). The functional importance and particular roles of these clusters for the translational apparatus are still poorly understood. For the three Fe/S clusters in the human mitoribosome, a structural role has been hypothesized, including the compensation for rRNA deletions and facilitation of protein interactions (6). Recent cell biological studies indicated an important role of the mS25-bS16m protein pair in redox-sensitive Fe/S cluster binding and mitoribosome assembly and function (19, 20).

The presence of Fe/S clusters in the mitoribosome links two important functions of mitochondria, *de novo* Fe/S cluster assembly (21) and mitochondrial translation (12). Intriguingly, human mitochondrial translation indirectly involves two other Fe/S cluster-containing enzymes, that is, the mitochondrial tRNA methylthiotransferase CDK5RAP1 containing two [4Fe-4S] clusters (22) and the recently described methyltransferase-like [4Fe-4S] protein METTL17 (yeast Rsm22) (23) which was shown to function in SSU assembly. Fe/S clusters are synthesized in mitochondria by the bacteria-derived, multiprotein iron-sulfur cluster assembly (ISC) machinery, consisting of a core set of early-acting ISC proteins required for *de novo* [2Fe-2S] cluster synthesis, trafficking, and apoprotein insertion, along with late-acting ISC proteins specifically involved in [4Fe-4S] cluster synthesis and insertion (Fig. 1*A*; for detailed reviews see (21, 24, 25, 26)). Fe/S cluster insertion into the mS25-bS16m protein pair also depended on the ISC system (19, 20).

**Figure 1:**
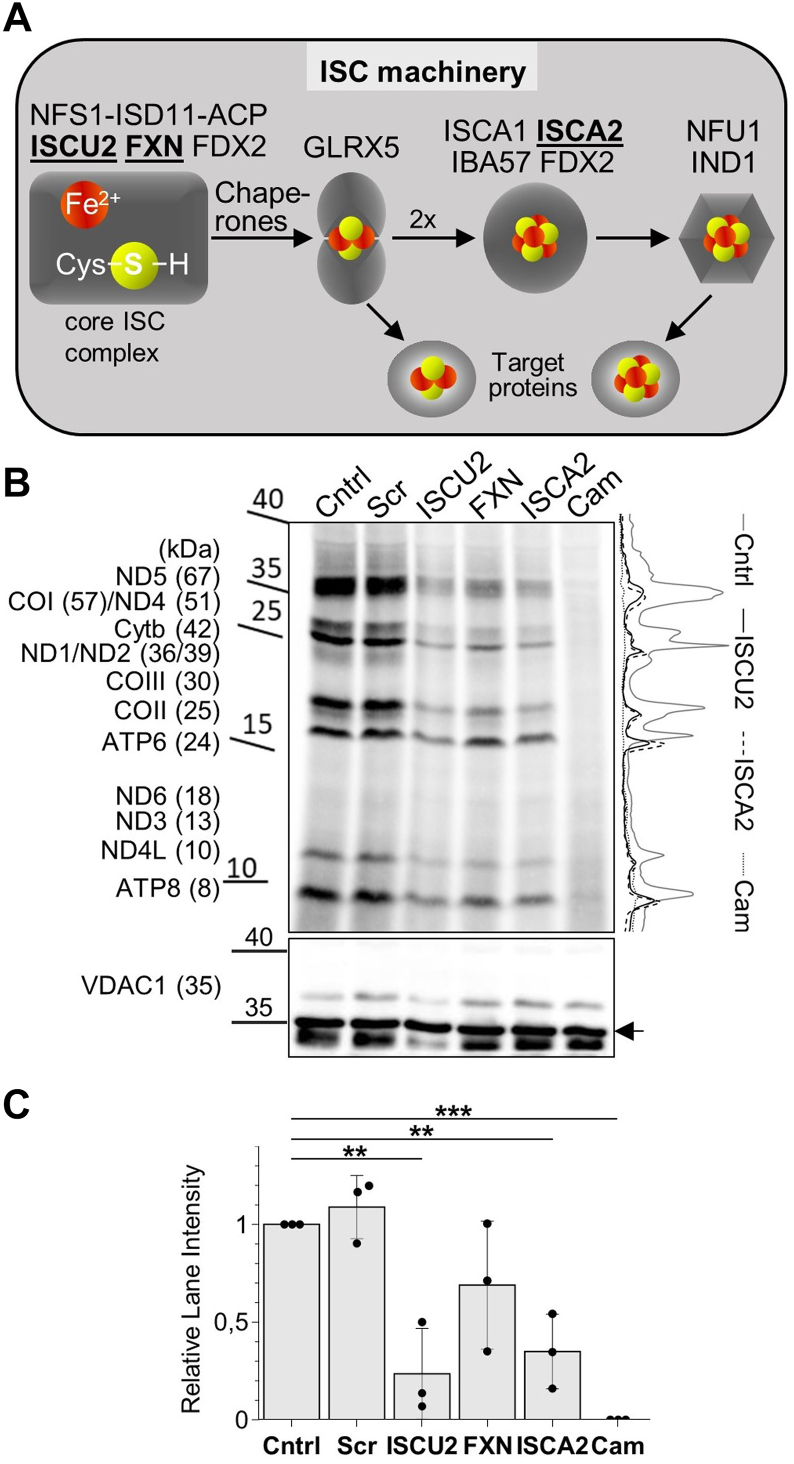
**Depletion of early and late ISC factors hampers mitochondrial translation.***A*, overview of human ISC biogenesis factors involved in [2Fe-2S] and subsequent [4Fe-4S] cluster formation and delivery to target proteins (21). The ISC proteins employed here are in *bold*. *B,* HeLa cells were mock-transfected (Cntrl), transfected with scrambled siRNA (Scr), or transfected with pools of siRNA against ISCU2, FXN, or ISCA2 for depletion of these proteins by two consecutive transfections each followed by cell growth for 3 days. After six days, cells were radiolabeled for 3 h in the presence of ^35^S-Met/Cys and the cytosolic translation inhibitor emetine to specifically radiolabel mtDNA gene products. As a negative control, cells were treated with the mitochondrial translation inhibitor chloramphenicol (Cam). Whole cell lysates were prepared and proteins separated by SDS-PAGE, followed by Western blotting and visualization of labeled translation products by autoradiography (*top*). Immunostaining of VDAC1 served as loading control (*bottom*). A representative autoradiography and immunostaining experiment is shown (n = 3 biological replicates). Observed molecular masses (kDa) are indicated. *C*, the average intensities of the radioactivity quantitated in each lane (examples are shown on *right part of B*) as mean ± SD (relative to Cntrl; set to 1) were calculated from three independent experiments and compared to Cntrl samples, with *p* values obtained from “one-way repeated measures ANOVA” and “Bonferroni *post hoc*” tests (∗∗*p* < 0.01 and ∗∗∗*p* < 0.001). ISC, iron-sulfur cluster assembly; mtDNA, mitochondrial DNA; VDAC1, voltage-dependent anion channel.

To explore the physiological role of all three ribosome-bound [2Fe-2S] clusters and to address the aforementioned linkage between mitochondrial Fe/S cluster assembly and translation, we aimed i) to verify that mitochondrial protein translation depends on the function of the mitochondrial ISC machinery, ii) to study the cell biological effects of Fe/S cluster-deficient mitoribosomes on, for example, their assembly and translation efficiency as well as on respiratory function, and iii) to *in silico* define those groups of eukaryotic species that contain these mitoribosomal [2Fe-2S] clusters.

## Results

### Both the early and late ISC components impact mitochondrial protein translation

The presence of three [2Fe-2S] clusters in the high-resolution cryo-EM structure of the human mitoribosome (6) suggested a requirement of the mitochondrial ISC machinery for ribosome function in translation. Initially, we expected the core (early-acting) ISC proteins rather than the late-acting ISC proteins to be essential for these functions, since only the former factors are needed for [2Fe-2S] cluster assembly (27, 28) (Fig. 1*A*). To test this idea, we measured the mitochondrial protein synthesis activity *via* ^35^S-methionine/cysteine (Met/Cys) radiolabeling of HeLa cells, in which early (ISCU2 and FXN) or late (ISCA2) ISC factors were depleted by treatment with established pools of siRNA (27, 29, 30). The cytosolic protein synthesis activity was specifically inhibited by emetine. SDS-PAGE and membrane blotting of whole-cell extracts followed by autoradiography revealed that all mitochondrial ^35^S-labeled translation products were diminished in all three ISC factor-depleted samples, as compared to cells transfected with no siRNA (Cntrl) or scrambled siRNA (Scr) (Fig. 1, *B* and *C*). Addition of chloramphenicol (Cam), an inhibitor of mitochondrial translation, served as a control to confirm the specific labeling of mitochondrially translated proteins. In line with earlier findings for the efficiency of the three RNAi treatments, the ISCU2 depletion had the strongest effect on mitochondrial protein translation efficiency (up to 5-fold decrease), while the effect upon FXN depletion was weaker (27, 31, 32, 33).

The results clearly revealed the expected requirement of mitochondrial Fe/S protein biogenesis for mitoribosomal translation. However, also the depletion of ISCA2, a protein specific for [4Fe-4S] protein assembly (27), led to a strongly reduced mitochondrial translation efficiency, even though the mitoribosome contains only [2Fe-2S] clusters. This observation may be explained by the function of the recently described mitochondrial [4Fe-4S] protein METTL17 which has been shown to bind to the mitoribosomal SSU and to be important for mitoribosomal assembly (23). In addition, a lack of the two [4Fe-4S] clusters in the mitochondrial [4Fe-4S] protein CDK5RAP1 involved in tRNA modification may contribute to the observed decreased translation efficiency (see Discussion) (22). Collectively, our results clearly show that mitochondrial protein synthesis capacity depends on active mitochondrial Fe/S protein biogenesis. However, a definitive conclusion on the functional relevance of the three mitoribosomal [2Fe-2S] clusters cannot be drawn by this approach, because [4Fe-4S] cluster-containing mitochondrial proteins may precede the function of the [2Fe-2S] clusters within the mitoribosome.

### Mitoribosomes can be radiolabeled with ^55^Fe

To directly explore the presence of Fe/S clusters in the mitoribosome, we employed an established ^55^Fe radiolabeling-immunoprecipitation procedure for detection of *in vivo* Fe/S protein assembly (34). For specific affinity precipitation of the SSU, HEK293 cells producing a doxycycline-inducible FLAG-tagged mS27 were used (35). The cells were simultaneously depleted for the two SSUs, mS25 and bS18m, by repeated transfection with specific siRNA at a 3-day interval or mock-treated (Fig. S1). After the second transfection, ^55^Fe-labeled transferrin was added to the medium, and a day later the expression of FLAG-tagged mS27 was induced with doxycycline. After a total siRNA depletion time of 6 days (including cell growth in the presence of ^55^Fe for 3 days and mitoribosomal incorporation of mS27-FLAG for 2 days), mitochondria-enriched cell fractions were prepared and lysed. SSU and monosomes (*i.e.* SSU and LSU) were precipitated using an anti-FLAG resin. As a control to monitor the nonspecific pull-down of ^55^Fe, cells inducibly expressing mitochondria-targeted Su9-FLAG-TEV-EGFP-PEST were transfected (Cntrl), and analyzed in the same way as the mS27-FLAG cells.

**Figure 2:**
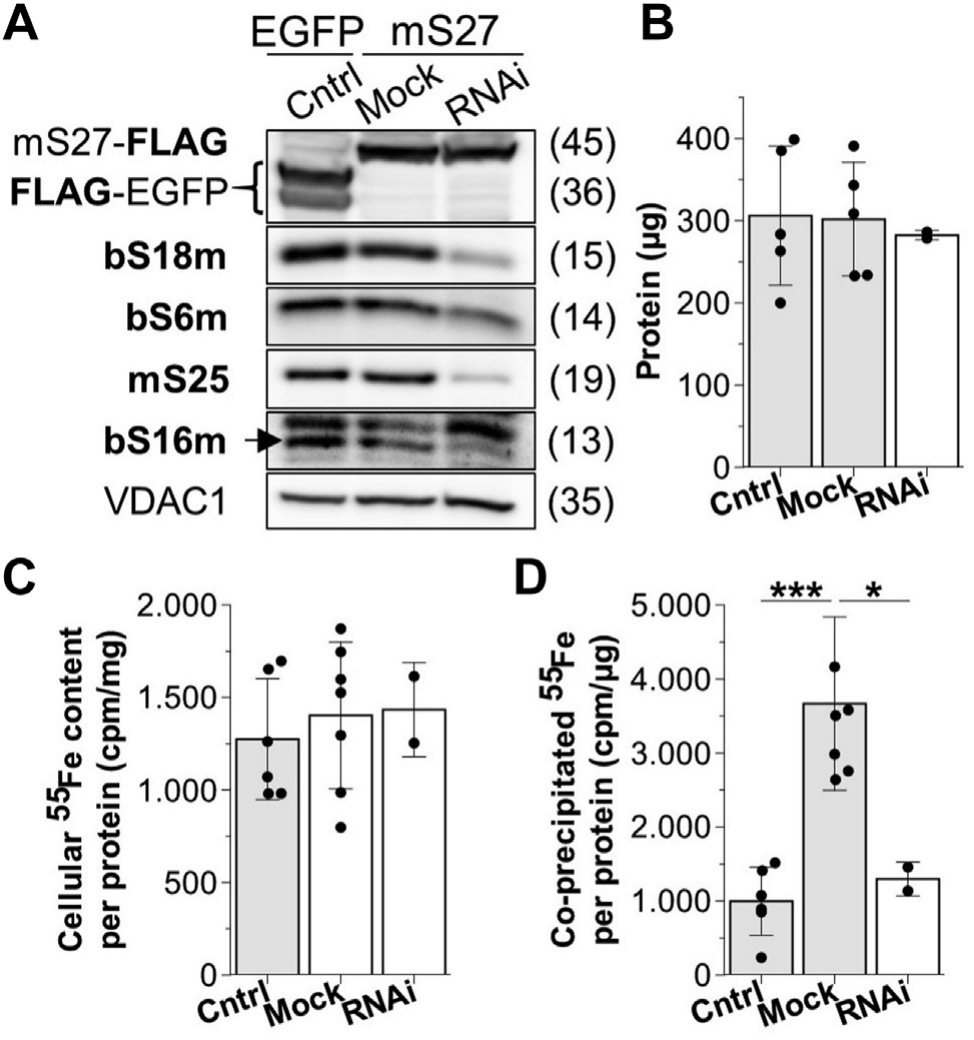
^**55**^**Fe precipitation by mS27 MRP responds to bS18m/mS25 double depletion.** HEK293 Flp-In TRex cells inducibly expressing C-terminally FLAG–tagged mS27 MRP (mS27-FLAG) were siRNAi-depleted for both bS18m and mS25 for a total of 6 days (RNAi), or mock-transfected. Cells inducibly expressing Su9-FLAG-TEV-EGFP-PEST (FLAG-EGFP) instead of mS27-FLAG were mock-transfected and served as negative control (Cntrl). Depletion was achieved by two consecutive rounds of siRNA transfection at a 3 day interval, and after the second transfection ^55^Fe-labeled transferrin was added to the culture medium (Fig. S1*A*). One day later, expression of mS27-FLAG or Su9-FLAG-TEV-EGFP was induced by doxycycline. *A*, cells were harvested and levels of indicated proteins were visualized by immunoblotting of total lysates. See Fig. S1*B* for controls. Observed molecular masses (kDa) are indicated. *B*, protein recovery 3 days after the second transfection per 1 × 10^6^ seeded cells. *C*, the ^55^Fe content of total cell lysates was analyzed by scintillation counting and related to total protein yield. *D*, the ^55^Fe content of anti-FLAG immunoprecipitates was analyzed by scintillation counting and related to protein amounts used for immunoprecipitation. Values of biological replicates are given as mean ± SD (n = 2 for RNAi and n ≥ 5 for Cntrl and mock), with *p* values obtained from one-way repeated measures ANOVA and Bonferroni *post hoc* tests (∗*p* < 0.05; ∗∗∗*p* < 0.001). MRP, mitochondrial ribosomal protein.

Simultaneous depletion of both mS25 and bS18m by RNAi was efficient as estimated by Western blotting and resulted in a concomitant decrease of the respective mitoribosomal partner proteins bS16m and bS6m (Fig. 2*A*). In contrast, several other mitochondrial proteins (*e.g.*, Rieske Fe/S protein, SDHB, NDUFV2, and VDAC1) were not affected (Fig. S1*B*). The total protein yield in the three cell culture samples was similar, as was the cellular ^55^Fe content as estimated by scintillation counting of cell extracts (Fig. 2, *B* and *C*). The FLAG-affinity precipitate of mock-treated, mS27-FLAG–expressing cells contained a significant amount of ^55^Fe, and was 3-fold higher than the background signal obtained from EGFP-expressing control cells (Fig. 2*D*). Importantly, combined depletion of mS25 and bS18m decreased the amount of mS27-associated ^55^Fe to background levels, indicating a specific ^55^Fe labeling of the SSU. We further attempted to evaluate the dependence of ^55^Fe binding on members of the core ISC machinery, because they are responsible for [2Fe-2S] cluster assembly (21). However, upon core ISC protein depletion, cells accumulate considerable amounts of iron due to the induced iron starvation-like condition (36, 37, 38). As a consequence, we found increased amounts of ^55^Fe being recovered with the FLAG-affinity resin, possibly due to coprecipitation of iron aggregates. This effect prevented the analysis of core ISC-dependent ^55^Fe binding to the SSU. Nevertheless, our results show that mitoribosomes can bind ^55^Fe, likely as part of a Fe/S cluster.

### Alteration of the mitoribosomal Fe/S cluster-binding motifs impairs cell growth and affects mitochondrial morphology

**Figure 3:**
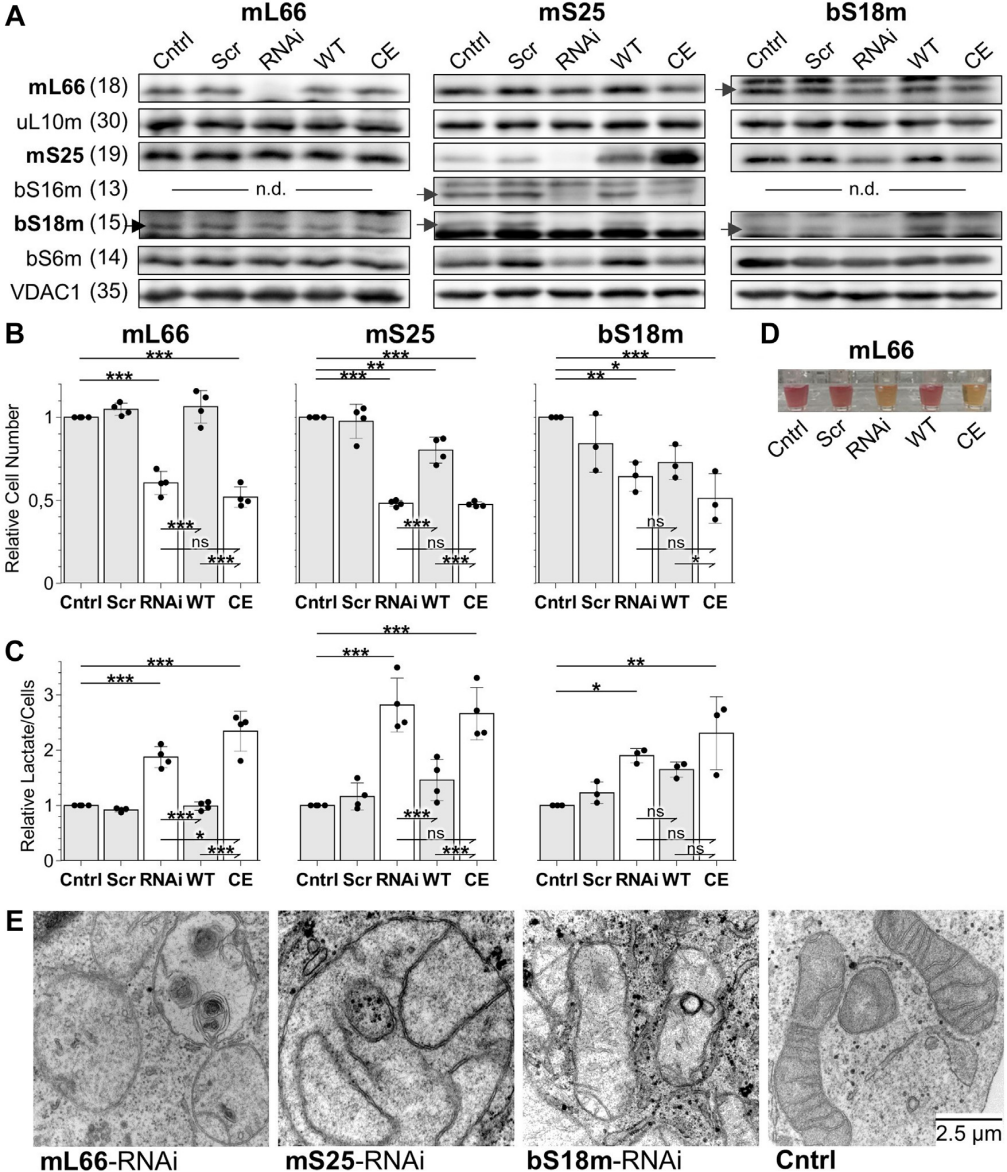
**Alteration of the Fe/S cluster-binding motifs of mL66, mS25, or bS18m leads to changes in mitochondrial morphology and increased lactate production.** HeLa cells were transfected twice at a 3-day interval without siRNA (Cntrl), scrambled siRNA (Scr), or pools of siRNA against mL66, mS25, or bS18m (RNAi) for depletion of these MRPs. Plasmids coding for the respective complementing WT proteins or Cys-exchange (CE) variants were included in RNAi transfections. *A,* at day 6, membrane-containing samples obtained from digitonin-based cell fractionation were used for immunoblotting against the indicated Fe/S cluster-coordinating MRPs, including VDAC1 as loading control. The RNAi-depleted and plasmid-complemented MRP subunits are in *bold*. n.d., not determined. *Arrows* indicate the specific protein bands. *B*-*C*, cell numbers at day 6 (*B*) and corresponding lactate concentrations in tissue culture supernatants per 10^6^ cells (*C*), both compared relative to Cntrl (set to 1), as means ± SD of 3 to 4 independent experiments, with *p* values obtained from one-way repeated measures ANOVA and Bonferroni *post hoc* tests. Test results among RNAi-treated samples are included. (∗*p* < 0.05; ∗∗*p* < 0.01; and ∗∗∗*p* < 0.001; ns, not significant). *D*, representative photograph of tissue culture supernatants from a mL66 depletion-complementation experiment at day 6. The *orange* to *yellow color* change of the phenyl red dye indicates acidification of the medium. *E*, representative electron micrographs showing abnormal mitochondrial morphology in cells depleted for mL66, mS25, or bS18m (Figs. S4 and S5). Mitochondria are enlarged, with fewer, sometimes onion-shaped cristae membranes in comparison to mitochondria from Cntrl cells. The scale bar (2.5 μm) applies to all images. Fe/S, iron-sulfur; MRP, mitochondrial ribosomal protein; VDAC1, voltage-dependent anion channel.

We designed an alternative strategy to explore the functional relevance of the three mitoribosomal [2Fe-2S] clusters, involving the exchange of Cys residues of the Fe/S cluster-binding motifs (also termed Cys motifs) and subsequent analysis of the physiological consequences. HeLa cell culture experiments were performed in which the MRPs mL66, mS25 or bS18m, each providing three Cys ligands, were depleted by repeated transfection with siRNA. In parallel, cells were complemented with plasmids coding for RNAi-resistant mRNAs of either the respective WT proteins or Cys-exchange (CE) variants lacking two or three of the Fe/S cluster ligands (mL66: C70A, C73A; mS25: C139S, C141S, C149S; bS18m: C65A, C68A). Subsequent Western blotting of pellet samples from a digitonin cell fractionation showed the successful depletion and efficient complementation of all three MRPs by the respective WT and CE proteins (Fig. 3*A*). The respective partner proteins were either unaffected by the manipulations (uL10m and bS6m) or followed the behavior of its partner (bS16m). The successful depletion and complementation allowed the investigation of the functional consequences.

HeLa cells siRNA-depleted for the mitoribosomal subunits mL66, mS25, or bS18m showed lower cell numbers (50–70%) than controls that were transfected without siRNA (Cntrl) or cells transfected with scrambled siRNA (Fig. 3*B*). The growth defect was associated with an acidification of the cell culture medium, and a 2-to 3-fold increase in lactate production (Fig. 3, *C* and *D*). This suggested that the siRNA-treated cells were strongly impaired in oxidative phosphorylation, and likely increased their glycolytic capacity. Complementation with WT mL66 and mS25 normalized cell growth and decreased the acidification and lactate concentrations to almost WT levels (Fig. 3, *C* and *D*). Strikingly, the respective CE variants neither reversed the growth defects nor the lactate levels suggesting that the Cys residues in mL66 and mS25 play an important function.

In contrast, no clear complementation effects were observed for bS18m (Fig. 3, *B* and *C*). Low bS18m expression as a reason for the failed complementation seemed unlikely from Western blots of bS18m, despite the weak signal detected in mitochondrial fractions (Fig. 3*A*, right). Further quantitative reverse transcription polymerase chain reaction measurements confirmed high levels of bS18m mRNA after plasmid-based expression and low levels upon siRNA depletion (Fig. S2). We also considered inefficient mitochondrial import of the bS18m precursor protein as a reason for failed complementation, because its native mRNA is predicted to contain a pumilio protein 2 (PUM2) recognition site for mRNA localization at the mitochondrial outer membrane (39, 40, 41). This motif was not included in our standard bS18m construct. We therefore performed the complementation assay with bS18m-encoding plasmids (WT and CE variant) that contain the native 3′-UTR including the PUM2 motif. Nevertheless, no significant improvement in bS18m protein levels and no complementation of the phenotypic effects were obtained. Moreover, the addition of a strong mitochondrial presequence (from *Neurospora crassa* Su9-ATPase) did not increase mitochondrial bS18m levels.

Upon depletion of any of the three MRPs, light microscopy revealed spherical and translucent vacuolar structures within the cells after six days of cultivation (Fig. S3). Similar observations had been made in a previous study upon depletion of the late ISC factors ISCA1, ISCA2, and IBA57 as a result of impaired Fe/S protein-dependent oxidative phosphorylation (27). In this study, electron microscopy had indicated cristae-deficient mitochondrial inner membranes. Similarly, electron microscopy of mL66-, mS25-, or bS18m-depleted cells showed abnormally enlarged mitochondria with a severe loss of cristae membranes, similar to respiratory-incompetent organelles (42, 43) (Figs. 3*E*; S4). The remaining mitochondrial inner membranes sometimes displayed an onion-like shape, reminiscent of cells depleted for the cysteine desulfurase NFS1 (36, 44). The matrix space showed only weak contrasting indicating rather low protein content. Quantification of the mitochondrial morphology revealed 58% of altered mitochondria in mL66-depleted cells (Fig. S5). These phenotypic effects could be reversed to WT morphology by complementation with the WT mL66 protein, but not the respective CE variant. Together, the results show that the Fe/S cluster-binding motifs of mL66 and mS25 are important for cell growth, mitochondrial function, and morphology. For bS18m, the scenario seems similar, but definitive proof for such notions would require successful phenotypical complementation by the WT protein.

### Alteration of the mitoribosomal Fe/S cluster-binding motifs impairs respiratory complex function

**Figure 4:**
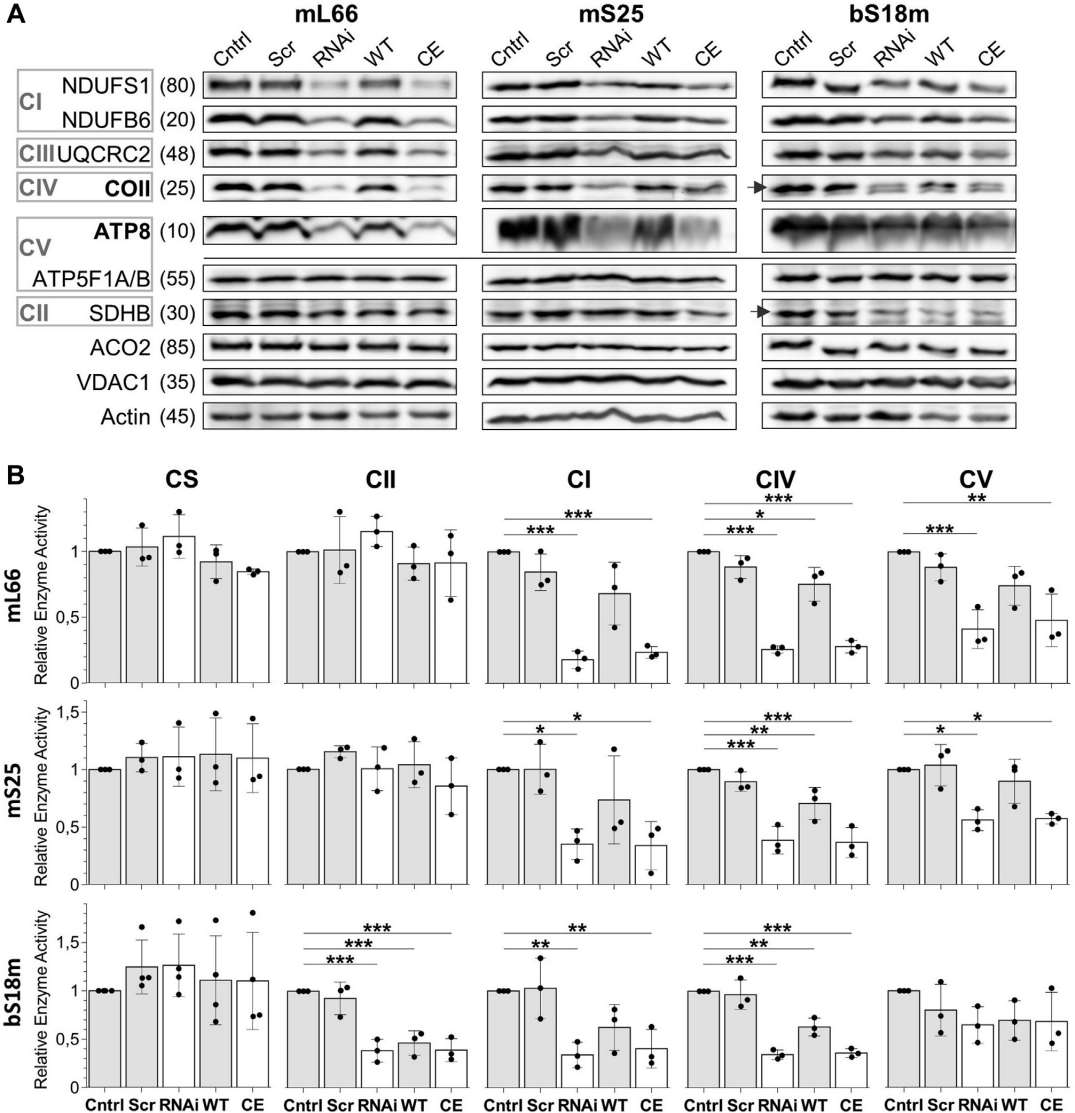
**Alteration of the Fe/S cluster-binding motifs of mL66, mS25, or bS18m decreases levels and activities of distinct respiratory chain complexes.** HeLa cells were treated and complemented as described in Figure 3. *A*, digitonin membrane fractions were used for immunostaining of the indicated protein subunits of respiratory complexes I to V (CI–CV). mtDNA-encoded subunits are shown in *bold*. Mitochondrial aconitase (ACO2) and VDAC1 as well as actin served as loading controls. Observed molecular masses (kDa) are indicated. *B*, activities of CI, CII, CIV, and CV were determined photometrically using mitochondria-enriched cell fractions, and were normalized to corresponding citrate synthase (CS) activities. *Bar graphs* show the mean ± SD of three independent experiments. Values were compared to Cntrl samples (set to 1), with *p* values obtained from one-way repeated measures ANOVA and Bonferroni *post hoc* tests (∗*p* < 0.05; ∗∗*p* < 0.01; and ∗∗∗*p* < 0.001). Fe/S, iron-sulfur; VDAC1, voltage-dependent anion channel; mtDNA, mitochondrial DNA.

Respiratory chain complexes I, III, IV, and V (CI, CIII, CIV, and CV), but not complex II (CII) contain mitochondrial DNA (mtDNA)-encoded subunits, and thus functionally depend on mitoribosomal translation. Hence, the depletion-complementation approach was employed for estimating the protein levels and activities of these complexes. Immunoblotting of membrane fractions prepared from digitonin-permeabilized mL66-, mS25-, or bS18m-depleted HeLa cells showed a diminution of mtDNA-encoded subunits of CIV and V, that is, COII and ATP8, respectively (Fig. 4*A*). Further, nucleus-encoded subunits of CI and CIII (NDUFS1, NDUFB6, and UQCRC2) were depleted. Apparently, the assembly of the whole complexes is hampered in the absence of mtDNA-encoded subunits, and the remaining subunits become degraded (33). In case of mL66 and mS25, efficient complementation of these effects by the respective WT proteins, but not by the CE variants clearly showed the importance of the mitoribosomal Fe/S proteins and particularly their Cys motifs for respiratory chain integrity. In contrast, protein levels of the αβ subunits of CV (ATP5F1A/B) were not altered, consistent with the observation that they can form a soluble F_1_ particle independently of mtDNA gene expression (45). The levels of CII subunit B (SDHB), or mitochondrial aconitase (ACO2), both being nucleus-encoded Fe/S proteins, were not or only slightly reduced (Fig. 4*A*). The abundance of further control proteins, for example, the voltage-dependent anion channel of the mitochondrial outer membrane or cytosolic actin, remained unchanged by the manipulations.

Next, the enzyme activities of the CI, CII, CIV, and CV were measured in isolated mitochondria (Fig. 4*B*). Consistent with the respective respiratory chain subunit levels (see above), in cells depleted of the ribosomal subunits mL66 and mS25, the activities of CI, CIV, and CV were severely diminished to 35%, 25%, and 50%, respectively, of WT levels. Complementation by the respective WT proteins restored the activities to almost normal, yet expression of the CE variants was without any significant beneficial effects, clearly showing the importance of the Cys motifs of mL66 and mS25 for the enzymatic functions of these respiratory complexes. As expected, due to the lack of mtDNA-encoded subunits, the activity of CII was not significantly affected, as was the activity of the control enzyme citrate synthase (Fig. 4*B*).

Similarly, depletion of bS18m by a pool of siRNA led to decreases in protein levels and enzyme activities of CI and CIV, yet in this case neither complementation with the WT bS18m nor with the CE variant was able to clearly rescue the effects. Another difference of bS18m depletion to both mL66 and mS25 was the only weak diminution of CV activity and an unexpected decrease of CII activity, along with lower protein levels of SDHB (Fig. 4, *A* and *B*). Also, these effects could not be complemented. To exclude off-target effects by the RNAi treatment, the different siRNA within the bS18m-directed pool were tested individually, and additionally a set of three different siRNA from another supplier were examined. However, all siRNA that effectively depleted bS18m levels also decreased SDHB protein levels. At this point, we do not have a satisfying explanation for this strong, likely indirect effect on CII. Notably, in bacteria a complex of S18 and S6 ribosomal proteins regulates their expression by binding to their own mRNAs (46, 47). A similar regulatory role on the translational level, or later upon membrane translocation and assembly might exist in mammals, and might interfere with complementation of bS18m protein depletion.

Taken together, these results indicate that the mitoribosomal subunits mL66, mS25, and likely also bS18m are essential for the integrity and activities of mtDNA-dependent respiratory chain complexes. For mL66 and mS25 our data also convincingly show that their Fe/S cluster-binding motifs are crucial for these functions. These findings demonstrate the importance of the mitoribosomal [2Fe-2S] clusters for mitochondrial protein translation and subsequently for mitochondrial respiration.

### Alteration of the mitoribosomal Fe/S cluster-binding motifs of mL66 and mS25 impairs mitochondrial protein translation

**Figure 5:**
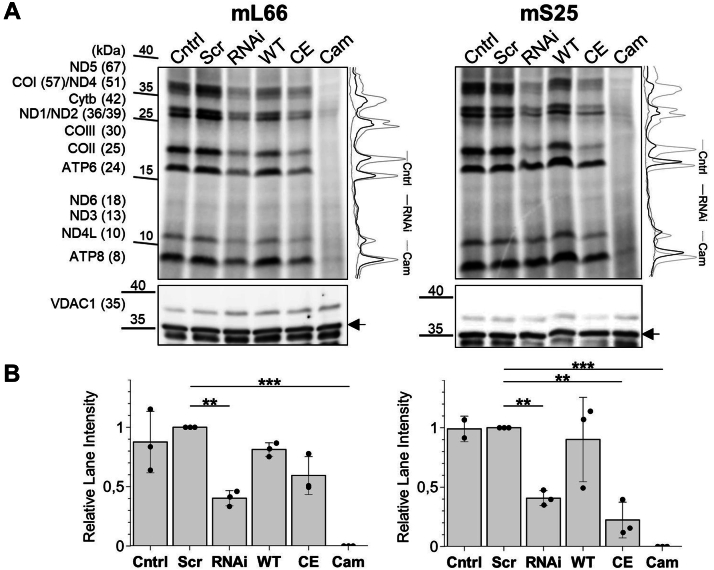
**The Fe/S cluster-binding motifs of mL66 and mS25 are crucial for *de novo* mitochondrial protein synthesis.** HeLa cells were treated and complemented as described in Figure 3. Radiolabeling with ^35^S-Met/Cys and autoradiography were performed as described in Figure 1. Data for bS18m depletion-complementation are shown in Fig. S6. *A*, representative autoradiographs showing the indicated mitochondrial translation products (*top*) and immunostaining of VDAC1 serving as loading control (*bottom*). Observed molecular masses (kDa) are indicated. *B*, quantitation of lane intensities for mL66 and mS25 (see *right parts of A* for examples), given as mean ± SD of three independent experiments, and presented relative to Scr values (set to 1); *p* values were calculated from one-way repeated measures ANOVA and Bonferroni *post hoc* tests (∗∗*p* < 0.01 and ∗∗∗*p* < 0.001). Fe/S, iron-sulfur; VDAC1, voltage-dependent anion channel.

To directly test the dependence of mitochondrial translation on mL66, mS25, and bS18m MRPs and their Fe/S cluster-binding motifs, ^35^S-Met/Cys radiolabeling of mtDNA-encoded proteins was performed (*cf.* Fig. 1*B*), and the newly synthesized mitochondrial proteins were analyzed by autoradiography and quantitation (Fig. 5). Upon siRNA depletion of mL66 or mS25, mitochondrial translation was severely decreased (by ca. 60%) compared to control cells. Complementation with the mL66 and mS25 WT proteins normalized the translation efficiency, while the mL66 and mS25 CE variants only weakly or not at all restored translation. This finding clearly shows the importance of the Cys motifs of mL66 and mS25 for mitoribosome function. Overall, these results for *de novo* synthesized mtDNA-encoded proteins fit well to the decreased steady-state levels of COII and ATP8 as detected by immunostaining (*cf.* Fig. 4*A*). Similar to our findings above, bS18m depletion and complementation yielded no conclusive change in mitochondrial protein synthesis activity and hence was not followed further (Fig. S6).

### The mitoribosomal Fe/S cluster-binding motifs are required for ribosome assembly

**Figure 6:**
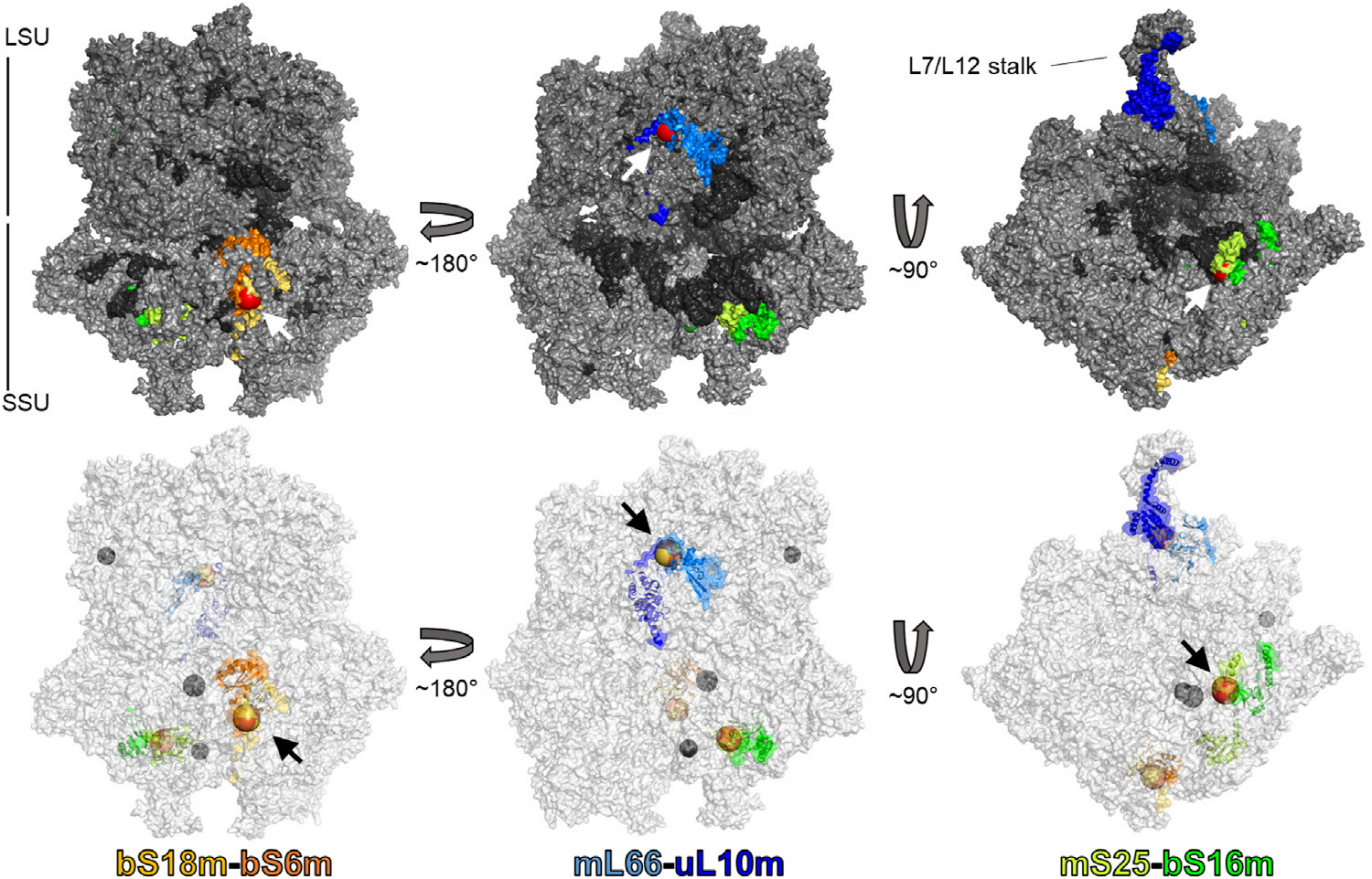
**Position of the three [2Fe-2S] clusters in the human mitoribosome.** Surface representations of the human mitoribosome, illustrating the peripheral, surface-exposed positions of the three [2Fe-2S] clusters and their coordinating MRPs. *Upper images* show the surface of proteins (*gray*) and rRNA (*dark gray*) with the Fe/S cluster-coordinating protein pairs colored in *yellow-orange, cyan-blue, and light-dark green* as indicated. The Fe/S clusters are shown in *red*. *Bottom images* present the transparent surface of mitoribosomes, with Fe/S cluster-coordinating protein pairs as *cartoon* in indicated colors. The Fe/S clusters (*arrows*) are depicted as *orange-yellow spheres*, the three Zn^2+^ ions as *dark gray spheres*. Images are based on structure PDB: 6zm6 (5). Fe/S, iron-sulfur; MRP, mitochondrial ribosomal protein.

**Figure 7:**
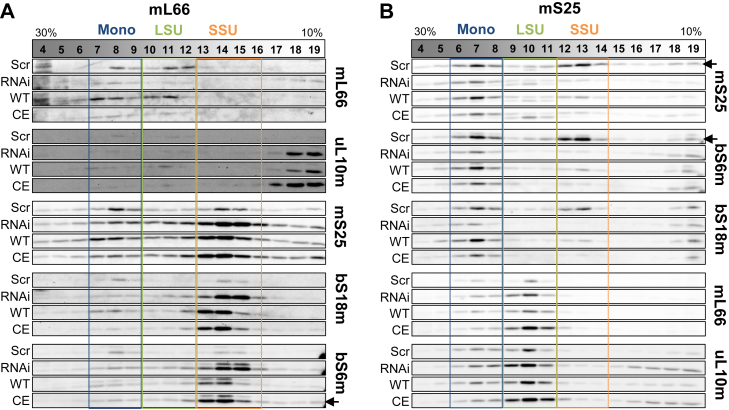
**A role of the Fe/S cluster-binding motifs of mL66 and mS25 in mitoribosome assembly and/or stability.***A*-*B*, HeLa cells were treated with siRNA and plasmids to deplete and complement for the subunits mL66 (*A*) and mS25 (*B*), as described in Figure 3. Mitochondria were isolated, lysed, and proteins were separated by centrifugation on 10 to 30% sucrose density gradients. Fractions were collected; proteins were precipitated with MeOH-CHCl_3_ and analyzed by immunoblotting for the indicated mitoribosomal proteins. *Arrows* indicate the specific signals for the MRPs. (Note: *the lower band* in mS25 blots belongs to mL66, sometimes visible due to preceding staining. Similarly, the *upper band* in bS6m blots originates from previous bS18m staining). Results from similar bS18m depletion experiments are shown in Fig. S8. Immunostainings for control proteins ATP5F1A/B and SDHB are presented in Fig. S7. Scr, scrambled control siRNA; RNAi, siRNA-mediated depletion; WT, complementation of siRNA-mediated depletion by wild-type proteins; CE, complementation of siRNA-mediated depletion by Cys-exchange variants; Fe/S, iron-sulfur; MRP, mitochondrial ribosomal protein.

The results presented so far indicate that the Fe/S cluster-binding motifs of mL66 and mS25 are essential for mitoribosomal translation. This raised the question of which particular function the [2Fe-2S] clusters may play in the complex translational process. Possibilities include a role in MRP folding or ribosomal subunit assembly which both may depend on the bridging function of two MRPs jointly binding the [2Fe-2S] cluster. Further, an active role in the translational process cannot be excluded, even though this may seem less likely because all three [2Fe-2S] clusters are located more in the periphery rather than in the functional core of the ribosomal subunits (Fig. 6). To get insights into this question, we analyzed the consequences of depleting the mL66 or mS25 and their replacement by the respective WT proteins or CE variants on mitoribosome assembly. Mitochondria were isolated from cells depleted and complemented as described above (*cf.* Fig. 3), lysed, and analyzed by sucrose density gradient centrifugation. Gradient fractions were collected, and the precipitated proteins were separated on SDS-PAGE and immunostained for selected MRPs (Fig. 7). ATP5F1A/B and SDHB served as control proteins and were virtually unaffected by the treatments (Fig. S7).

In control cells treated with scrambled siRNA the mL66 protein was found in both LSU and monosome fractions (Fig. 7*A*). The siRNAi treatment depleted mL66 as observed above. Complementation with WT mL66 gave a similar protein distribution as for Scr cells, showing the successful assembly and incorporation of plasmid-expressed mL66 into both LSU and monosome. On the contrary, complementation with the CE variant resulted in only little mL66 protein in LSU and monosome fractions, comparable to the siRNA-treated sample. The CE variant therefore may not assemble properly into the LSU. Since hardly any mL66 CE variant was observed in monomer fractions, the unassembled CE variant, which is detectable before centrifugation (*cf.* Fig. 3*A*), might have become degraded or aggregated during the long centrifugation-fractionation period. Immunostaining of the LSU protein uL10m, the Fe/S cluster-binding partner of mL66, showed the protein in monosome and LSU fractions, even though staining was weak (Fig. 7*A*). Upon RNAi-mediated depletion of mL66 and complementation with mL66 WT or CE proteins, the weak immunoblot signal for uL10m in monosome and LSU fractions did not differ significantly from the control (Scr), indicating that this protein can assemble into the mitoribosome independently of its mL66 partner protein. Under siRNA treatment conditions, a relatively high portion of uL10m was observed in monomeric fractions, as evident from all three biological replicates and particularly under conditions with nonfunctional mitoribosomes. A possible explanation could be that uL10 m expression is upregulated upon siRNA-mediated loss of mL66.

Staining of SSU proteins mS25, bS18m, and bS6m showed their predominant presence in monosome and SSU fractions (Fig. 7*A*). Their amounts in the SSU relative to monosome fractions were strongly increased upon mL66 RNAi treatment, as compared to control cells (Scr), again indicating an siRNA effect on other mitoribosomal proteins. In contrast to RNAi and CE samples, complementation with WT mL66 shifted bS18m and mS25 proteins to some extent into monosome fractions, suggesting a restored capacity to form monosomes from LSU and SSU particles by WT mL66 protein and not the CE variant.

The sucrose density gradient analysis of mitoribosomes obtained from analogous mS25 RNAi depletion and complementation experiments unveiled effects on both SSU and LSU particles, reminiscent of the combined LSU- and SSU-specific effects upon mL66 depletion (Fig. 7*B*). In Scr control samples, mS25 and the SSU protein pair bS18m-bS6m were present mainly in monosome and SSU fractions, as expected. Upon mS25 siRNA treatment, this protein was severely decreased, yet more predominantly in SSU than in monosome fractions. This effect could efficiently (for monosomes) or partially (for SSU) be complemented by expression of WT mS25, but not the CE variant despite its strong overproduction (*cf.* Fig. 3*A*, middle). Apparently, the mS25 CE variant could not be incorporated into the SSU, and/or was prone to proteolysis or aggregation during centrifugation-fractionation, similar to mL66 CE (see above). Interestingly, upon mS25 siRNA treatment (RNAi, WT, and CE samples), the amounts of the SSU proteins bS18m and bS6m were also mainly decreased in SSU relative to monosome fractions, and were restored by complementation with the WT mS25 but not the CE variant. The results align well with the lower abundance of bS18m and bS6m proteins in mS25-depleted mitochondria (*cf.* Fig. 3*A*). The overall lower amount of the three SSU proteins may indicate a disturbed SSU formation. We conclude that upon mS25 deficiency the formation and/or stability of the entire SSU (and accordingly the monosome) was affected. Strikingly, these effects could not be compensated by the mS25 CE variant.

In conspicuous analogy to the accumulation of SSU proteins upon mL66 depletion, the amounts of mL66 and uL10m were increased in LSU relative to monosome fractions in samples depleted for mS25RNA (mainly RNAi and CE samples), in comparison to control cells (Scr; Fig. 7*B*). In contrast, hardly any alterations in both mL66 and uL10m levels were found in monosome fractions of all mS25-treated samples. However, the severely diminished translational activity of mS25-depleted mitoribosomes detected in the ^35^S-radiolabeling experiment (Fig. 5) suggested that these monosomes were not or only partially functional.

Finally, upon bS18m depletion, the pattern of immunodetected MRPs in the sucrose density gradient fractions closely resembled the mS25 depletion experiment (Fig. S8). The levels of bS18m were diminished in both SSU and monosome fractions, and could be partially complemented by WT bS18m, but not CE variant. Concomitantly, the other two SSU proteins (mS25 and bS6m) were less abundant clearly indicating an impaired SSU assembly and, in turn, decreased monosome formation, as in case of mS25 depletion. In contrast, the LSU proteins mL66 and uL10m were hardly changed in LSU fractions in all samples of the bS18m depletion experiment, while they were reduced in monosome fractions of bS18 siRNA-treated samples (RNAi, WT, and CE), consistent with a decreased amount of functional SSU for monosome formation.

In summary, the sucrose density gradient experiments suggest an impaired assembly and/or stability of the LSU or SSU, when the MRPs mL66 or mS25 and bS18m, respectively, were depleted. The Cys motifs of mL66, mS25, and bS18m were crucial for this effect, since the CE variants could not support the formation of stable LSU or SSU particles, and consequently no functional monosomes could be assembled.

### Phylogenetic distribution of the Fe/S cluster-binding motifs in mitoribosomes

**Figure 8:**
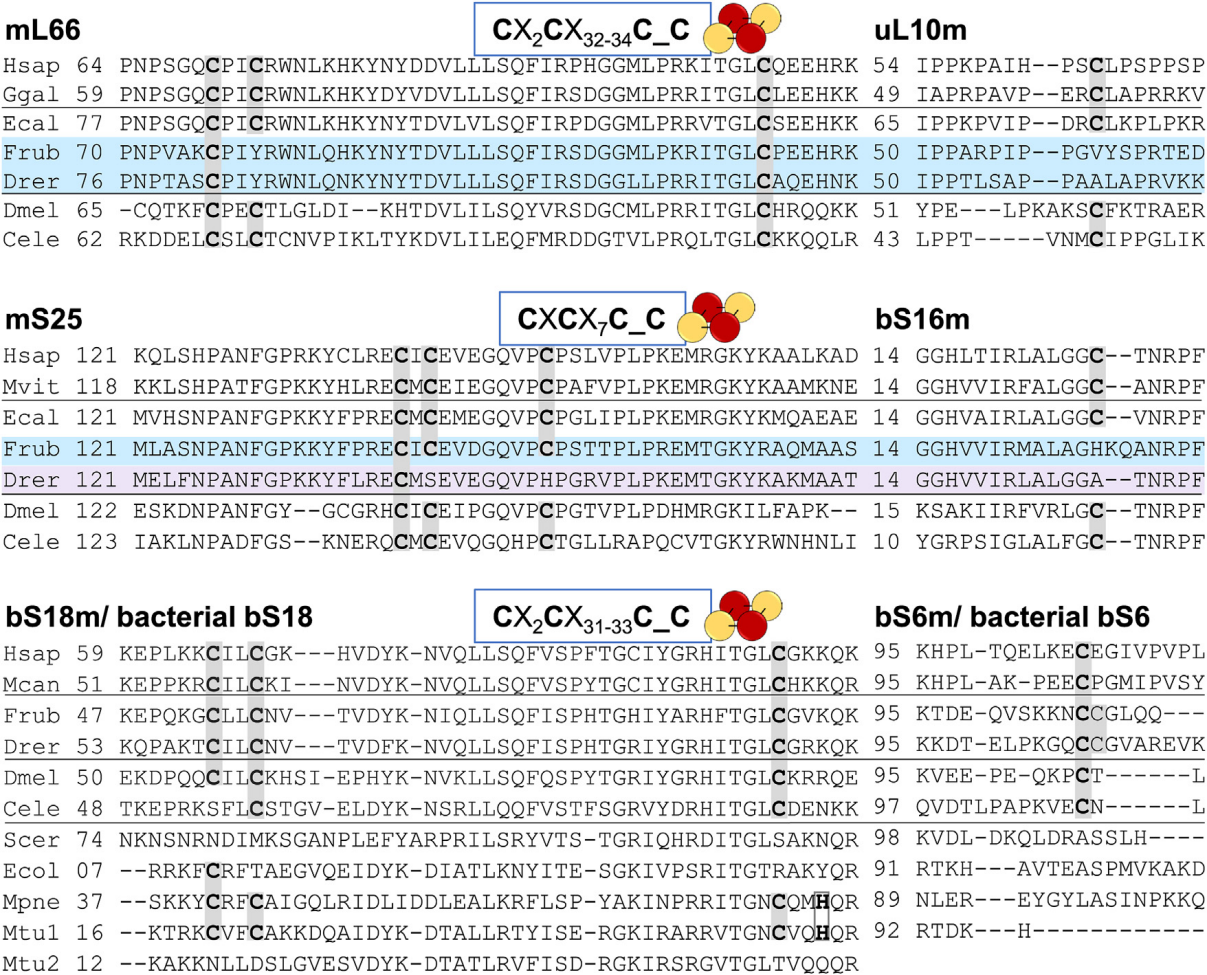
**Conservation of Fe/S cluster-binding Cys residues in mL66, mS25, bS18m, and their respective partner proteins.** Multi-sequence alignments of mitoribosomal proteins from *Homo sapiens* (Hsap); birds: *Gallus gallus* (Ggal), *Manacus vitellinus* (Mvit), *Manacus candei* (Mcan); fish: *Erpetoichthys calabaricus* (Ecal), *F. rubripes* (Frub), *D. rerio* (Drer); other: *D. melanogaster* (Dmel); *C. elegans* (Cele); *S. cerevisiae* (Scer). For comparison, bacterial ribosomal proteins related to the bS18m-bS6m pair are presented for *Escherichia coli* (Ecol), *M. pneumoniae* (Mpne), and *M. tuberculosis* (Mtu1, Mtu2). *Gray boxes* highlight Cys residues that coordinate the [2Fe-2S] clusters in the human mitoribosome (6). The conserved Cys motifs of the three MRP pairs are given in boxes above the sequence. In contrast to reedfish (Ecal) and other ancient fish species, modern ray-finned fish (*e.g.*, *F. rubripes* or *D*. *rerio*, *light blue*) do not possess the conserved the Cys motifs. The bacterial ribosomal protein RpsR (orthologous to bS18m) from *M. pneumoniae* and one of the paralogs from *M. tuberculosis* contain a Zn-finger motif (CX_2_C…CX_2_H), which is conserved among several bacteria (see Table S1) and binds a Zn^2+^-ion as indicated by ribosome cryo-EM structures (51, 52). Fe/S, iron-sulfur; MRP, mitochondrial ribosomal protein.

As mentioned above, present-day eukaryotic mitoribosomes are characterized by a large structural variation (16). The mitochondria-specific MRPs mS25 and mL66, that in humans coordinate two of the three mitoribosome [2Fe-2S] clusters, are present in most metazoans, (Fig. S9). On the contrary, bS18m, bS6m, and bS16m are considered to be of alpha-proteobacterial origin, and accordingly have homologs in the phyla of both Eukarya and Bacteria. Finally, the universally present MRP uL10m is found also in all three domains of life (48). Intriguingly, ribosomal protein sequences differ substantially among different species, and this particularly applies to the Fe/S cluster-binding motifs. For instance, bS18 proteins in, for example, *Saccharomyces cerevisiae, T. thermophilus*, and *Escherichia coli* are lacking the Fe/S cluster-binding motif present in the human MRP. We therefore aimed to better understand the phylogenetic distribution of the Cys motifs that potentially coordinate mitoribosomal Fe/S clusters in eukaryotes. To this end, multisequence alignments were generated for protein relatives of mL66, mS25, and bS18m, as well as their respective partner proteins uL10m, bS16m, and bS6m. The results were analyzed for the presence of the Cys motifs (Fig. 8 for representative species; Figs. S10–S12 for deeper analysis).

The multisequence alignment of mL66 proteins from 217 vertebrates revealed that the Cys motif **C**X_2_**C**X_32-34_**C** (bold letters indicate (potentially) Fe/S cluster-coordinating residues) is highly conserved in mammals, birds, reptiles, amphibians, some species of ancient fish (but not other fish) and, slightly modified, also in *Drosophila melanogaster* and *Caenorhabditis elegans* (Figs. 8, S10). In striking similarity to mL66, the fourth Cys ligand for Fe/S cluster coordination provided by uL10m is conserved in these species suggesting that they, like humans, contain a [2Fe-2S] cluster in the mL66-uL10m protein pair. In the alignment (Fig. S10), the ancient species of fish containing the fully conserved Cys motif in the mL66-uL10m pair comprise, for example, lamprey (*Petromyzon marinus*), cartilaginous fish (great white shark (*Carcharodon carcharias*), elephant shark (*Callorhinchus milii*)), and some primitive species which split early in evolution from the modern ray-finned fish, namely reedfish (*Erpetoichthys calabaricus*), asian bonytongue (*Scleropages formosus*) and *Paramormyrops kingsleyae* (49, 50). In all other analyzed modern ray-finned fish (*e.g.*, Osteoglossocephalai, including the puffer fish *Fugu rubripes*), the mL66 motif was altered to a conserved **C**PIYX_34_**C** sequence, lacking the second conserved Cys. Strikingly, in these species the fourth Cys ligand in the uL10m partner protein was also absent (Fig. S10), making it highly likely that these species do not contain a [2Fe-2S] cluster in this protein pair.

Next, multi-sequence alignments of mS25- and bS16m-orthologs were analyzed for the presence of the conserved Cys motif **C**X**C**X_7_**C**_**C** (underscore indicates separation of the two MRPs) coordinating a [2Fe-2S] cluster in human mitoribosomes (Figs. 8, S11). Conservation of these four Cys residues is found in mammals, birds, reptiles, amphibians, some ancient fish, as well as *D. melanogaster* and *C. elegans*. The identified ancient types of fish with the fully conserved Cys motif in the mS25-bS16m pair are elephant shark, hagfish (*Eptatretus burgeri*) or lamprey, coelacanth, and the early diverged species of ray-finned fish reedfish, spotted gar (*Lepisosteus oculatus*), asian bonytongue and *P. kingsleyae*. As observed for other MRPs, some organisms were found to contain more than one paralog of mS25. These included marine mammals and a few species from Cnidaria and Porifera, and usually at least one of the paralogs contained the full Cys motif. Similar to the Cys motif in mL66-uL10m, modern ray-finned fish showed deviations from this motif. In species of the large Euteleostei clade (*e*.*g*., in *F. rubripes*) the single Cys in bS16m is replaced by histidine, leading to a **C**X**C**X_7_**C**_**H** motif, which in principle still could hold a Fe/S cluster. In Otocephala, the sister clade to Euteleostei, encompassing zebrafish (*Danio rerio*) or channel catfish (*Ictaluarus punctatus*), two Cys of mS25 were replaced by serine (Ser) and histidine (His) residues, and in bS16m alanine (Ala) was present instead of Cys. These alterations result in a conserved **C**XSX_7_**H**_A motif that is unlikely to coordinate a [2Fe-2S] cluster.

Finally, multisequence alignments of the bS18m protein, which is of bacterial origin and widely abundant, unveiled a **C**X_2_**C**X_31-33_**C** motif that is conserved in several eukaryotic species, for example, mammals, birds, fish, nonvertebrates and insects, but not in bikonts or fungi (Figs. 8 and S12). More prevalent than observed for mS25 or mL66 (see above), many vertebrates encode multiple paralogs of bS18m, usually at least one containing all three conserved Cys. The fourth Cys ligand provided by bS6m is conserved in species in which the bS18m Fe/S cluster-binding partner contains the Cys motif. The simultaneous conservation of the Fe/S cluster-coordinating Cys residues in the bS18m-bS6m pair (**C**X_2_**C**X_31-33_**C**_**C** motif) suggests the likely presence of a bound [2Fe-2S] cluster in these species. For completeness, bacterial bS18 proteins were also aligned. Interestingly, diverse phyla of Bacteria contained a conserved motif (**C**X_2_**C**X_n_**C**X_2_**H**) in the region homologous to the Cys motif in bS18m. For instance, this motif was found to be abundant among Terrabacteria, but virtually absent in Pseudomonadota including Alphaproteobacteria (Table S1). Structures of the ribosome of *Mycobacterium tuberculosis* or *Mycoplasma pneumoniae* contain a Zn^2+^ ion bound by this motif (PDB: 7MT3 (51), and PDB: 7OOC (52)). Similar to bS18m paralogs in vertebrates *M. tuberculosis* possesses two paralogous S18 proteins, one with (RpsR1) and one without (RpsR2) the Cys motif.

In summary, multisequence alignments of orthologs of the human [2Fe-2S] cluster-binding MRPs showed high conservation of the Cys motifs in the mL66-uL10m, mS25-bS16m, and bS18m-bS6m protein pairs of most vertebrates. It therefore seems likely that the mitoribosomes of these species also contain all three [2Fe-2S] clusters present in the human mitoribosome. Unexpectedly, the Cys motifs of mL66-uL10m and mS25-bS16m are not conserved in modern ray-finned fish, where Cys residues are replaced by His, Tyr, Ser, or nonligating residues. This renders the presence of mitoribosomal Fe/S clusters in these species unlikely.

## Discussion

Fe/S proteins are known to be involved in numerous cellular processes (21). Nevertheless, in recent years several new Fe/S proteins have been identified, some of which were previously known as Zn-binding proteins (53, 54). A prominent example is the human mitoribosome, for which high resolution structures identified three metal-binding sites to contain [2Fe-2S] clusters (6). This observation was even more surprising, because Fe/S clusters are not known as a common feature of bacterial ribosomes, the evolutionary ancestors of the mitoribosome (8). The finding suggested an intimate connection between mitochondrial Fe/S protein biogenesis and protein translation. Here, we performed cell biological and biochemical experiments with human cultured cells to verify the presence of all three [2Fe-2S] clusters in the mitoribosome, test their biological importance, and gain initial information about their functional role. For one of these clusters, bound to the mS25-bS16m protein pair, excellent work published during the preparation of our article, has provided ample evidence for a structural role of this cluster (19). Further, a requirement of the mitochondrial ISC system for Fe/S cluster insertion and a possible function of the cluster as a redox sensor were documented (19, 20). In our work, we used additional methods and analyzed the morphological consequences upon loss of each of the three Fe/S clusters by electron microscopy. Finally, we employed bioinformatic analyses to determine the phylogenetic distribution of the three mitoribosome [2Fe-2S] clusters within the eukaryotic kingdom.

We initially determined the dependence of mtDNA-encoded protein translation on the process of Fe/S protein assembly assuming that the [2Fe-2S] clusters in the mitoribosome should depend on early- but not late-acting ISC factors (27, 28). We found, however, a clear requirement of both early (ISCU2 and FXN) and late (ISCA2) ISC factors for mitochondrial translation, a result that does not readily indicate the necessity of the [2Fe-2S] type of clusters in the mitoribosome. An explanation for this unexpected finding was provided by the recently published methyltransferase-like METTL17 that contains a [4Fe-4S] cluster, and was shown to assist mitoribosome assembly (55). The precise mechanism of its action is still ill-defined. A further, yet more indirect connection between mitochondrial Fe/S protein biogenesis and translation may be the tRNA-modifying CDK5RAP1 protein, which carries a radical SAM [4Fe-4S] cluster (22). *CDK5RAP1* KO mice display an age-related hearing loss, yet are viable suggesting at least residual activity of mtDNA-encoded translation (56). In fact, KO cells maintained mitochondrial translation, yet at diminished level (22). The consequences of a cluster loss of CDK5RAP1 on translation have not been studied. Overall, these intricacies precluded any conclusions about the dependence of mitochondrial translation on the mitoribosomal Fe/S cluster types (*i.e.* [4Fe-4S] or [2Fe-2S]) from these ISC protein depletion experiments.

We therefore used an established ^55^Fe radiolabeling affinity-purification method (30) to directly explore Fe/S cluster binding to mitoribosomes. Affinity-purified small ribosomal subunits bound substantial amounts of ^55^Fe. Upon depletion of both mS25 and bS18m MRPs, coprecipitated ^55^Fe decreased to background levels consistent with the presence of Fe/S clusters on the SSU. Further assignment as [2Fe-2S] clusters by a dependence of ^55^Fe binding on early ISC factors was not possible, because early ISC factor-depleted cells (*e.g.*, ISCU2) show increased iron uptake and accumulate ^55^Fe aggregates (37, 38). This side effect apparently resulted in higher rather than diminished ^55^Fe coprecipitation. In contrast, depletion of several late-acting ISC factors, which do not impact iron regulation and cellular uptake, strongly decrease ^55^Fe binding to mitoribosomes (19). However, as outlined above, this effect likely is due to the [4Fe-4S] cluster-requiring METTL17 function in mitoribosome assembly. Hence, *via* ^55^Fe radiolabeling the binding of Fe to the mitoribosome could be confirmed, but the classical approach of ISC protein depletion to further discriminate between [4Fe-4S] and [2Fe-2S] clusters is not applicable to the mitoribosome.

As an alternative experimental approach, we employed a protein depletion-complementation strategy in which cultured human cells were first RNAi-depleted of the Fe/S cluster-binding MRPs mL66, mS25, or bS18m. Then, cells were complemented with either the respective WT proteins or variants of the Fe/S cluster-binding motif exchanging Cys for Ala or Ser residues to impede Fe/S cluster assembly. This approach allowed us to evaluate the physiological importance of both the Fe/S cluster-binding mitoribosome protein pairs and their respective Cys motifs. Our results clearly show that each of the three Fe/S cluster-coordinating protein pairs is necessary for normal cell growth as well as for WT levels and enzymatic functions of mtDNA-encoded respiratory complexes, but not of the nucleus-encoded complex II as a control. This MRP requirement is remarkable given the rather peripheral localization of these protein pairs within the ribosome particles, emphasizing the importance of peripheral MRPs. All these phenotypic effects are readily explained by the observed strong defects of MRP-depleted cells in mitochondrial translation. Further, MRP depletions elicited severe alterations in mitochondrial morphology. Enlarged organelles with a low-density matrix space and the lack of cristae membranes closely resemble previously described examples of respiratory-incompetent or mtDNA-deficient mitochondria (37, 42, 43). These RNAi-depletion effects were specific for mL66 and mS25 deficiencies, as evident from the successful complementation by respective WT proteins. In case of bS18m, complementation was rather weak. Moreover, RNAi depletion of bS18m led to a decrease in complex II activity which also could not be complemented. The molecular reason for these unexpected side effects remains unknown.

In contrast to the successful complementation of mL66- and mS25-depleted cells by the respective WT proteins, Cys-motif variants were unable to revert the above mentioned biochemical phenotypes including the defects in mitochondrial translation. Likewise, the morphological changes of mitochondria were not complemented by the mL66 variant. The lack of significant complementation in case of bS18m (see above) did not allow conclusions for this protein’s Fe/S cluster-binding motif. Collectively, the results document, at least for the mL66-uL10m and mS25-bS16m protein pairs, that their Fe/S cluster-binding motifs are crucial for mitoribosome function and the observed downstream effects. We conclude that the two bound Fe/S clusters are essential cofactors of the human mitoribosome.

The MRP-depleted and WT- or Cys-motif variants-complemented cells were further used to purify mitochondria and to analyze detergent extracts by density gradient centrifugation for the abundance of mitoribosomal particles. These analyses showed that either MRP-depleted or Cys-motif variants were impaired in the assembly of the respective LSU or SSU, and hence the formation of functional monosome particles. Complementation with the WT MRPs normalized the effects. The nonimpaired ribosomal subunit was even increased in amounts, suggesting a compensating effect. The ribosome subunit assembly defects provide a mechanistic explanation for the diminished translation efficiency, leading to the respiratory chain defects and the observed morphological phenotypes. As further shown by immunostaining, the Cys variants of mL66 and mS25 were efficiently produced and translocated to mitochondria, but apparently the final incorporation into functional ribosomal subunits and monosomes did not take place. Hence, the Cys residues and their bound Fe/S clusters appear to be essential for proper folding of the MRP pairs and their stable assembly on the ribosomal particles by interaction with other MRPs and/or rRNAs. Interestingly, clinical cases with mutations in human bS16m or mS25, yet unrelated to the Cys motifs, were reported (57, 58). The observed alterations cause problems in the folding of the MRPs and lead to defects in SSU and monosome assembly similar to the ones observed in our study for the Cys motif. This highlights the importance of a single MRP for the whole translational machine.

Interestingly, our *in silico* searches revealed a distribution of the three Fe/S cluster-binding Cys motifs in all vertebrates with the exception of modern ray-finned fish, which encompasses the majority of known fish. In ray-finned fish, the Cys motif in the mL66-uL10m protein pair is modified to **C**PIY[X_34_]**C**_A/V, and the mS25-bS16m Cys motif among Otocephala is changed to **C**X**S**X_7_**H**_A. These modifications make Fe/S cluster binding, as observed in human or putatively other vertebrate mitoribosomes, rather unlikely. The Cys motif in mS25-bS16m of other fish (*i*.*e*., Euteleostei) is comprised of **C**X**C**X_7_**C**_**H**. A 3Cys-1His coordination could likely facilitate Fe/S cluster binding, even though with possibly altered redox properties. Only a few species, cartilaginous fish (Chondrichthyes) and more ancient clades of ray-finned fish retained the fully conserved Cys motifs. Given that the Cys motifs in mS25 and mL66 occur already in some Porifera and Cnidaria, and they are present in most other Protostomia and Sarcopterygii, the sister group of Actinopterygii, it seems likely that their common ancestor possessed them as well. In the process of evolution to modern fish (Teleosts), the Fe/S clusters may have been replaced, by internal amino acid interactions or the binding of Zn. These alterations may have occurred to accommodate to different environmental conditions like iron or zinc availability, reactive oxygen species or oxygen concentrations, or pH levels. The deviations from the canonical Cys motif within most ray-finned fish show that the crucial function of the Fe/S clusters in human mL66-uL10m and mS25-bS16m can be replaced by other structural solutions to preserve the functionality of mitoribosomes. These altered MRPs must maintain the ability to properly fold, interact with other MRPs and/or rRNA, and assemble into functional LSU and SSU even without the Cys motif. Since there is no fish mitoribosome structure available to date, the exact nature of these interactions is unknown. In species harboring Fe/S clusters in their mitoribosome, these cofactors may perform an additional regulatory role in that respiratory chain proteins are only translated when Fe/S cluster biogenesis is functional.

Some mitoribosomal proteins have paralogs and transcript variants that either contain or lack the Cys motif. This includes mS25 of marine mammals and bS18m with multiple copies in various vertebrates. Given the examples in fish, these variants may still allow the formation of functional ribosomes, despite the differences in amino acid sequences. Similar ideas have been controversially discussed in the context of the two bacterial S18 paralogs in *M. tuberculosis*, one of which binds Zn *via* a **C**X_2_**C**[…]**C**X_2_**H** motif (59, 60, 61, 62, 63). Currently, there is no information on the possible physiological relevance or function of these paralogs in higher vertebrates.

In summary, our results demonstrate that the three [2Fe-2S] clusters recently discovered in the human mitoribosome structure are essential cofactors for mitochondrial protein translation. The clusters represent important structural elements for proper folding and assembly of functional mitoribosomal subunits and the monosome. These cofactors, as studied in the human mitoribosome, are presumably present and function similarly in other higher eukaryotes containing the conserved Cys binding motifs.

## Experimental procedures

### Cell culture and transfection with siRNA and plasmids

HeLa cells were maintained at our institute and frequently tested for *Mycoplasma* infections. The cells were cultured in Dulbecco’s Modified Eagle Medium (DMEM, high glucose, GIBCO, Thermo Fisher Scientific) with standard supplementation of 7.5% fetal bovine serum (Advanced, Capricorn Scientific), 2 mM GlutaMAX, 100 IU/ml penicillin, and 100 μg/ml Streptomycin (complete DMEM). For transfection, cells were harvested by trypsinization, washed with electroporation buffer (20 mM Hepes, pH 7.1, 137 mM NaCl, 5 mM KCl, 0.7 mM Na_2_HPO_4_, 6 mM glucose (64)), counted and resuspended in electroporation buffer to a concentration of 7-10 × 10^6^ cells in 265 μl. For protein depletion experiments by RNAi, cell aliquots were mixed with either scrambled (Scr) control siRNA or a pool of target-specific siRNA (total concentration 2.26 μM; Table S2), 2 to 6 μg of expression plasmids (Table S3), and 3.75 μg of pVA-I plasmid to improve cell recovery (65, 66, 67). Electroporation was performed in a 4 mm cuvette with 265 V, 525 μF using a Xcell Gene Pulser (Bio-Rad). Subsequently, cells were cultured in the presence of 20% conditioned HeLa medium for 3 days. A second round of transfection was performed to achieve a total protein depletion time of 6 days.

HEK293 Flp-In TRex cells expressing C-terminally FLAG-tagged mS27 (mS27-FLAG) upon induction with doxycycline were a kind gift from Z. Chrzanowska-Lightowlers (35) and maintained in doxycycline-free complete DMEM. A HEK293 Flp-In TRex cell line inducibly expressing mitochondria-targeted Su9-FLAG-TEV-EGFP-PEST (harboring the presequence of Su9-ATP synthase from *N. crassa* and a destabilizing PEST sequence) served as control. Harvest by trypsinization and transfection by electroporation were carried out similar to HeLa cells using established protocols (68).

### siRNA and plasmids

Sets of three Silencer Select predesigned siRNA directed against the mRNA of mL66, mS25, or bS18m were purchased from Thermo Fisher Scientific. In addition, also two bS18m-directed Dharmacon ON-TARGET plus siRNA were used (Horizon Discovery LTD) (Table S2). Complementary DNAs encoding ribosomal subunits mL66, mS25, or bS18m were purchased from OriGene Technologies Inc. or Sino Biological Inc. (Table S3). The bS18m complementary DNA, received in a pCMV6-vector as FLAG-tagged fusion protein, was first cloned in a pCMV3-vector without a tag. Then, Cys exchange mutants were created for all three MRP subunits replacing 2 or 3 Fe/S cluster-coordinating Cys by Ala or Ser residues (Table S4). RNAi silent mutations were introduced to all six expression plasmids (WT and CE). All mutations were introduced by standard PCR mutagenesis techniques (69, 70) using primers listed in Table S4. To produce a transcript of bS18m including the 3′UTR with the PUM2 motif, the gene fragment was purchased from Twist Bioscience and cloned into the pCMV3 vector (see Table S3). To control for possible effects elicited by the overproduction of mitochondrially targeted MRPs, cotransfection of a plasmid encoding mitochondria-targeted Su9-DsRed2 (cloned from pDsRed2-C1, Takara Bio Inc; Table S3) was included in control transfections and siRNA transfections lacking complementation plasmids.

### ^35^S-Met/Cys radiolabeling of mitochondrially translated proteins

For analysis of newly synthesized mtDNA-encoded proteins, cells were transfected with siRNA and plasmids as described above. After the second round of transfection 0.4 to 0.6·× 10^6^ cells were cultured for another 3 days in duplicates in separate 6-well plates. After day six, control samples were treated with 75 μM Cam for 1 h to inhibit mitochondrial translation, and Cam was continuously maintained during all subsequent steps. Cells from one 6-well plate were then harvested for immunoblot analysis. The second set of cells was cultured for a total of 15 min in 1.5 ml Met- and Cys-free DMEM (Gibco) supplemented with glutamine and Pen/Strep (MCfree DMEM). Cytosolic translation was inhibited by adding 100 μg/ml emetine, and after 10 min 100 μCi/ml Met-[S35]-label (^35^S-labeled mixture of 70% Met and 25% Cys; Hartmann Analytic GmbH) were added for 3 h. Cells were then washed with MCfree DMEM, harvested by trypsinization, washed with ice-cold PBS, and dry cell pellets were stored at −80 °C until further use. Protein contents were determined *via* Bradford-Assay (Bio-Rad). Cells were solubilized in sample buffer, and 75 μg of whole cell protein was separated on a 13% tricine gel (71), followed by Western blotting. Radiolabeled proteins were visualized by phosphorimaging using a Sapphire Biomolecular Imager (Azure Biosystems). Total lane intensities were determined with supplied software, intensities of the Cam-containing sample were subtracted as background, and values were normalized to the Scr control sample.

### Cell fractionation and biochemical analyses

Preparation of crude mitochondria for subsequent enzyme activity assays or ribosome preparation was based on mechanical (plasma) membrane disruption. In brief, 8 to 10·× 10^6^ freshly harvested cells in 500 μl mito-buffer (25 mM Tris/HCl pH 7.4, 0.25 mM sucrose, 1.5 mM MgCl_2_, 1 mM PMSF) were homogenized with a motor-driven glass/Teflon homogenizer and centrifuged (10 min, 600*g*, 4 °C). Cell pellets were resuspended and homogenized a second time. Combined supernatants were centrifuged (20 min, 20,000*g*, 4 °C) to yield a soluble cytosolic fraction and a crude mitochondria-containing pellet. The pellet was resuspended in mito-buffer and used for subsequent enzyme activity assays for aconitase, complex II, citrate synthase, complex IV, and ATP synthase (CV) (29, 30, 72, 73). Complex I activity was determined as described (29) with the following modifications. The reaction buffer contained 12.5 mM Na_2_HPO_4_ pH 7.5, 0.375% bovine serum albumin, 2.5 mM MgCl_2_, 100 μM decyl ubiquinone, 210 μM NADH, and 1.73 mM KCN. The control sample for determination of background activity contained 17 μM rotenone. Absorption was recorded at 340 nm. Digitonin-based subcellular fractionation used for immunostaining of proteins was performed as described earlier (74).

### Immunoprecipitation of ^55^Fe-radiolabeled mitoribosomes

For the detection of mitoribosomal Fe/S clusters, cells were labeled with ^55^Fe according to established protocols (30). In brief, HEK293 mS27-FLAG or Su9-FLAG-TEV-EGFP-PEST cells were subjected to electroporation twice at a 3-day interval and subsequently cultured in presence of ^55^Fe-loaded transferrin for 3 days (Fig. S1*A*). On day 4, expression of FLAG-tagged mS27 was induced by addition of 2 μg/ml doxycycline. After harvest, mitochondria-enriched membrane fractions were prepared by digitonin-based cell fractionation, lysed in TNEGT buffer (10 mM Tris–HCl pH 7.4, 150 mM NaCl, 1 mM EDTA, 10% glycerol, 0.2% Triton X-100, and 1 mM PMSF), and subjected to anti-FLAG immunoprecipitation. The precipitate was analyzed for ^55^Fe radioactivity as a readout for [2Fe-2S] cluster content, and the ratio of recovered radioactivity per total protein input applied for immunoprecipitation provided a measure for the efficiency of Fe/S cluster incorporation into mitoribosomal subunits. Nonspecific pull-down of ^55^Fe was monitored by the analysis of mitochondrial fractions obtained from Su9-FLAG-TEV-EGFP-PEST cells.

### Lactate determination

Lactate in tissue culture medium was determined photometrically in a 96-well plate by combining lactate dehydrogenase (LDH)-based conversion of NAD^+^ to NADH/H^+^ and NADH/H^+^-dependent reduction of dichlorophenol-indophenol by diaphorase (75). Fresh medium and tissue culture supernatants were diluted 20-, 40-, and 60-fold in Hanks’ buffered salt solution (HBSS), and lactate standards from 0.125 to 2 mM in HBSS were prepared. Twenty microliters of each sample were mixed with 230 μl of reaction buffer containing 7 μM NAD^+^, 60 μM dichlorophenol-indophenol, 92 mU/ml diaphorase, and 1.84 U/ml LDH in HBSS. For the measurement of nonspecific background signals, diaphorase and LDH were excluded. The absorption at 600 nm was recorded for 30 min, and lactate concentrations were calculated from the specific reaction rates. Lactate amounts were corrected for the concentrations already present in fresh medium and normalized to the final cell number of the corresponding culture vessel with the control sample set to 1.

### Electron microscopy

HeLa cells transfected with siRNA and plasmids as described above were cultivated in 10 mm tissue culture dishes. Cells were washed twice with PBS, and fixation was done as described (76, 77). Postfixation was performed in 1% osmium tetroxide for 1 h at room temperature, followed by an overnight incubation with 0.3% uranyl acetate dissolved in 50 mM maleate buffer (pH 5.0). Samples were embedded in Epon according to standard procedures. Eighty nanometers thin sections were contrasted with lead citrate and uranyl acetate and were examined with a Zeiss EM 109S electron microscope equipped with a 1K × 1K CCD camera (Tröndle TRS). Images were taken using the ImageSP software (Sysprog, https://sys-prog.com/en/about).

To quantify defective mitochondria upon mL66 deficiency 60 pictures of each sample were taken. All mitochondria were counted, and those fulfilling at least two of the following criteria were considered as damaged: 1) less than three cristae related to size of cross section, 2) presence of abnormal cristae inner membranes, of cristae membranes with onion shape or of other mitochondria-internal structures, and 3) weak staining of matrix space.

### Separation of mitoribosome particles by sucrose gradient centrifugation

Separation of mitoribosomes was performed by sucrose gradient fractionation based on published protocols (12, 78). Briefly, crude mitochondria from ca. 20 × 10^6^ cells were resuspended in mitolysis-buffer (mito-buffer supplemented with 50 mM KCl, 18.5 mM MgCl_2_). cOmplete EDTA-free protease inhibitor mix (Roche) and 0.4 U/μl RNasin ribonuclease inhibitor (Promega) were added. Mitochondria were lysed by addition of 0.8% of Triton X-100, and the samples incubated for 15 min on ice. Lysates were cleared by centrifugation (20 min, 16,000*g*, 4 °C), loaded on a 10 to 30% sucrose gradient (prepared from a step gradient), and centrifuged in a SW41 rotor (16 h, 21,000 rpm, 4 °C). Fractions of 600 μl were collected and proteins were precipitated with MeOH-CHCl_3_, solubilized in 120 μl sample buffer, and 50 μl aliquots were subjected to SDS-PAGE followed by immunostaining.

### Gel electrophoresis and immunostaining

Cellular proteins were separated either by standard Tris-glycine SDS-PAGE using 6 to 20% acrylamide gradient gels, or by tricine SDS-PAGE (71). Proteins were transferred to nitrocellulose membranes and immunodetected using standard Western blotting techniques. Primary antibodies used for immunodetection are compiled in Table S5. Peroxidase-conjugated goat anti-rabbit (1:7500 dilution, Cat# 170-6515) and goat anti-mouse (1:2500 dilution, Cat# 170-6516) antibodies from Bio-Rad were used as secondary reagents. Chemiluminescence signals were recorded by an Intas ChemoStar imaging system.

### Alignments and phylogenetic trees

Orthologous protein sequences from vertebrates were retrieved from the Ensembl database (release 109, Feb 2023; (79)). Further sequences were retrieved from the NCBI database (www.ncbi.nlm.nih.gov/gene) using the *similar genes* search. Sequences of prokaryotic S18 ribosomal proteins were gathered from the microbial genome database (80). Multisequence alignments of proteins were done with Clustal Omega (81), and sequences were manually searched for the presence of conserved Cys motifs. PhyloTree files from Clustal Omega were used to create trees with iTOL (82).

## Data availability

Additional data can be found in the supporting information, complete species tree cladograms are available from the iTOL-server (link available on request), and electron microscopic images can be made available on request.

## Supporting information

This article contains supporting information. (27, 31, 33, 35, 43, 65, 68, 69, 80, 81, 82, 83, 84, 85)

## Conflict of interest

The authors declare that they have no conflicts of interest with the contents of this article.
