# Supplementary material for: Crucial role and conservation of the three [2Fe-2S] clusters in the human mitochondrial ribosome
Source: J Biol Chem. 2024 Dec 13;301(2):108087. doi: 10.1016/j.jbc.2024.108087 (PMC11791143; doi:10.1016/j.jbc.2024.108087)
Supplement: Supporting information [file mmc1.pdf]

Supporting information for

Crucial role and conservation of the three [2Fe-2S] clusters in the human mitochondrial ribosome

Linda Boß, Oliver Stehling, Hans-Peter Elsässer, and Roland Lill*

Institut für Zytobiologie im Zentrum für Synthetische Mikrobiologie SynMikro,
Philipps-Universität Marburg, Karl-von-Frisch-Str. 14, 35032 Marburg, Germany.

*Corresponding author:
Roland Lill
ORCID ID 0000-0002-8345-6518
Phone: +49-6421-286 6449
E-mail: lill@staff.uni-marburg.de

Content:

Supporting Figures S1-S12

Supporting Tables S1-S5

Supporting References

Supporting Figures

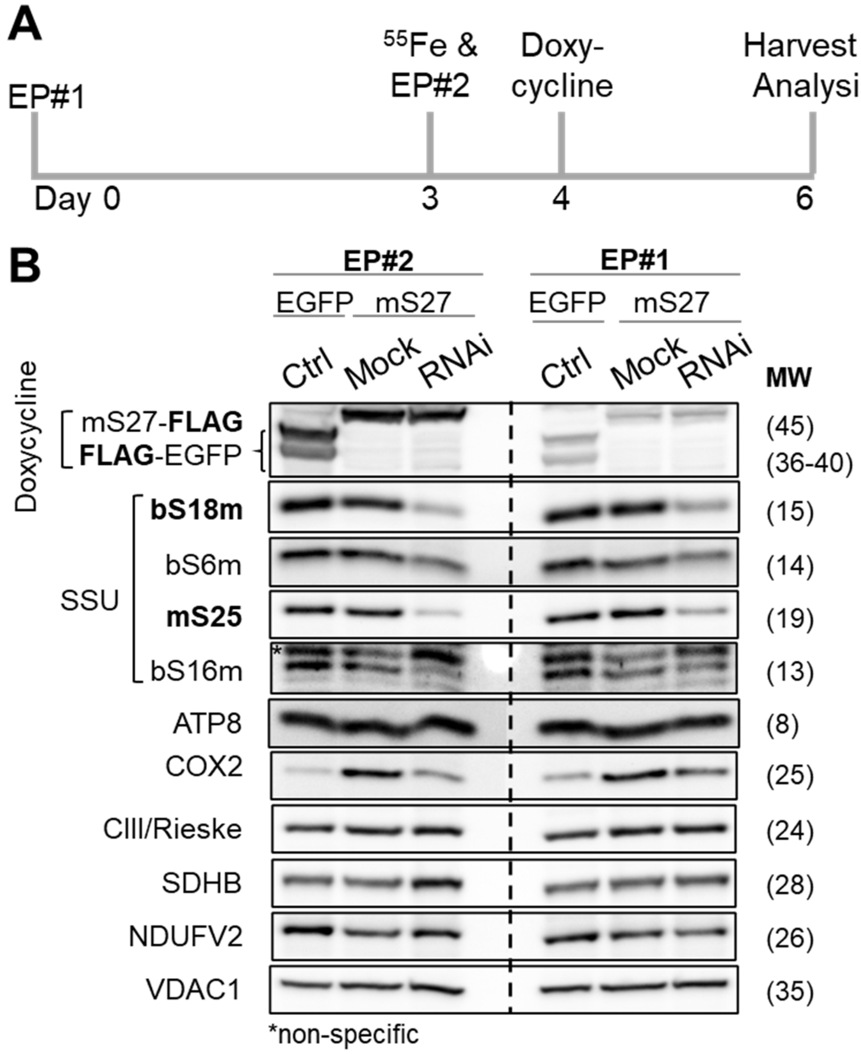

Figure S1. Phenotypic analysis of bS18m-mS25 double-depleted cells during ^{55}Fe radiolabeling of mitoribosomes. A. Timeline of the experimental setup of the radiolabeling experiment (cf. Fig. 2). HEK293 Flp-In TRex cells inducibly expressing C-terminally FLAG-tagged mS27 (mS27-FLAG) were first depleted for both bS18m and mS25 using specific siRNA pools by two consecutive transfections by electroporation (EP#1 and EP#2) for a total of 6 days. ^{55}Fe-labeled transferrin was added to the medium right after the second transfection. At day 4, mS27-FLAG and FLAG-EGFP synthesis were induced by doxycycline addition. After 3 and 6 days, cells were harvested for further analysis. **B.** Levels of the indicated proteins were visualized by immunoblotting of total cell lysates and compared to Mock transfected mS27-FLAG cells or control-transfected (Ctrl) Su9-FLAG-TEV-EGFP-PEST (FLAG-EGFP) cells. The blots from the upper left part are equivalent to Fig. 2A. The rest of the figure additionally shows the full data set for the entire experiment as outlined in A. Observed molecular masses (MW in kDa) are indicated. SSU, small mitoribosomal subunit.

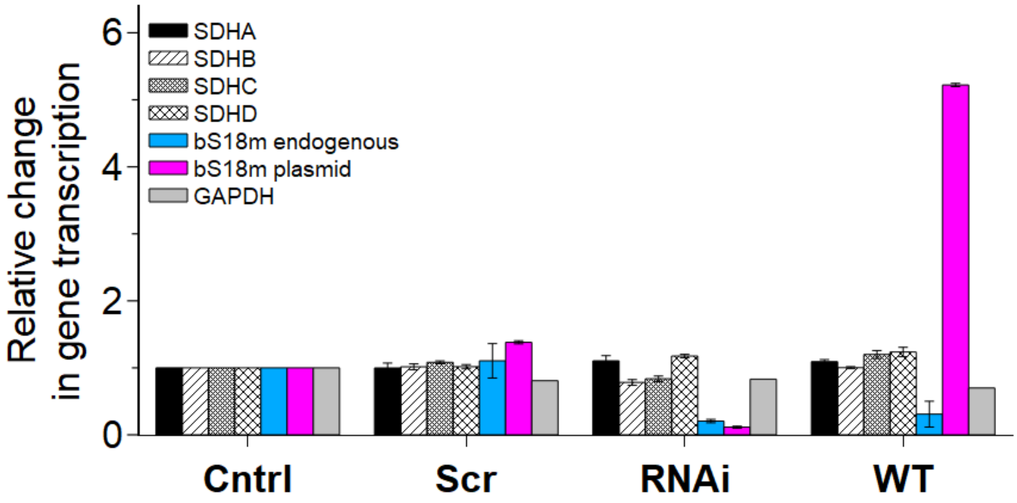

Figure S2. qRT-PCR analysis of SDH subunits expression upon RNAi depletion of bS18m. HeLa cells were RNAi-depleted for bS18m (RNAi) and complemented with wild-type bS18m (WT) via plasmid-based overexpression. Controls were either mock-transfected (Cntrl) or received scrambled siRNA (Scr). qRT-PCR was performed with primers targeting mRNAs of complex II (SDH) subunits A-D, endogenous bS18m or plasmid-based bS18m. GAPDH served as a control, and HPRT1 as internal reference. Presented $2^{-\Delta\Delta Ct}$ values were calculated from 3 technical replicates, ±SD values represent the intra-assay variability.

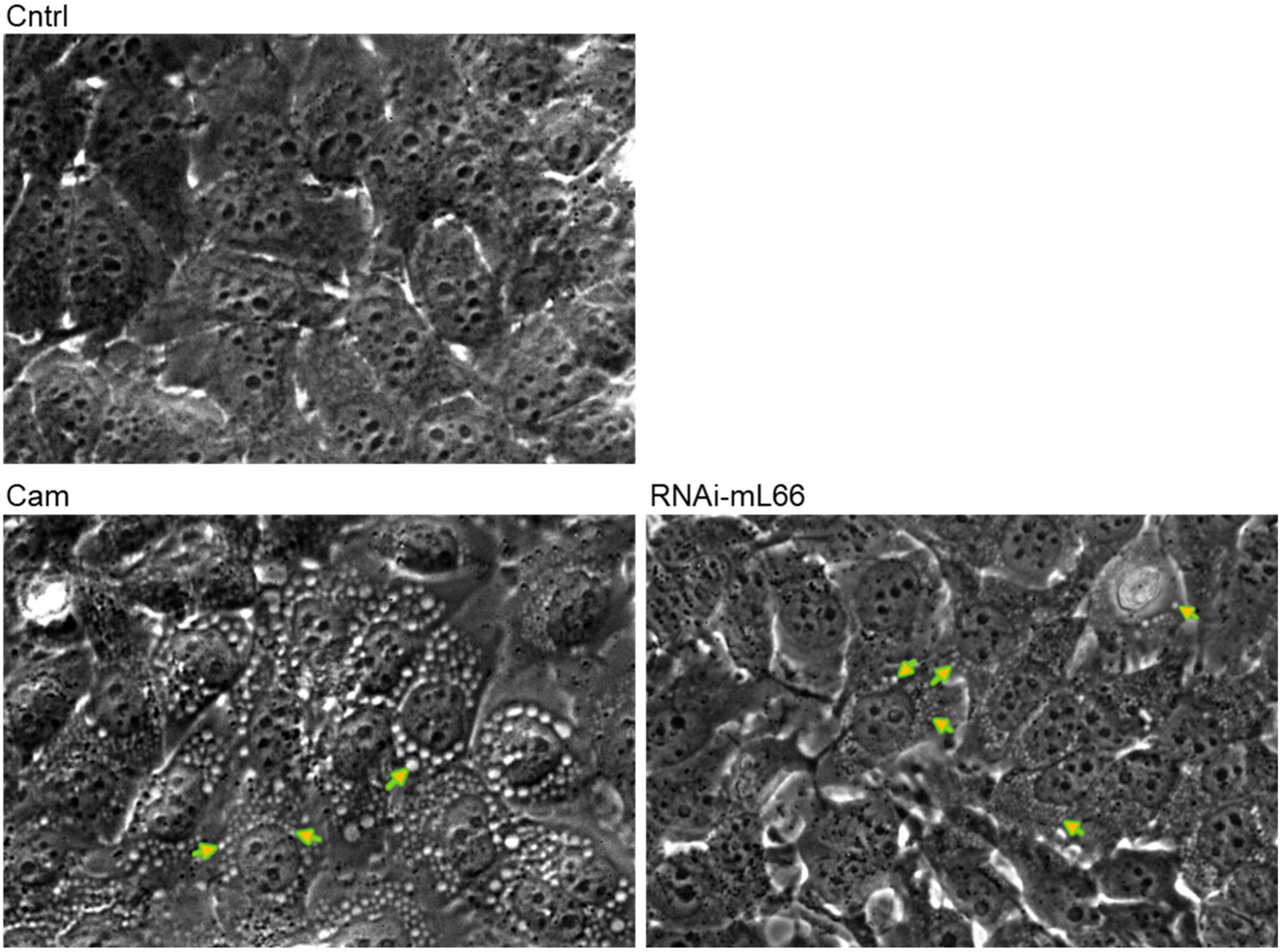

Figure S3. Depletion of mL66 in HeLa cells induces sponge-like vacuolar structures. HeLa cells were depleted for mL66 by two consecutive rounds of siRNA transfection as in Fig. 3 and visualized by phase contrast light microscopy after a total of six days of cultivation (RNAi-mL66). Bright vacuolar structures of differing size (examples indicated by arrows) were best visible in flat cells and around the nuclei. In a previous study, these structures were interpreted as enlarged mitochondria (27). Images of non-depleted HeLa cells (Cntrl) or HeLa cells treated with chloramphenicol (Cam) are shown for comparison.

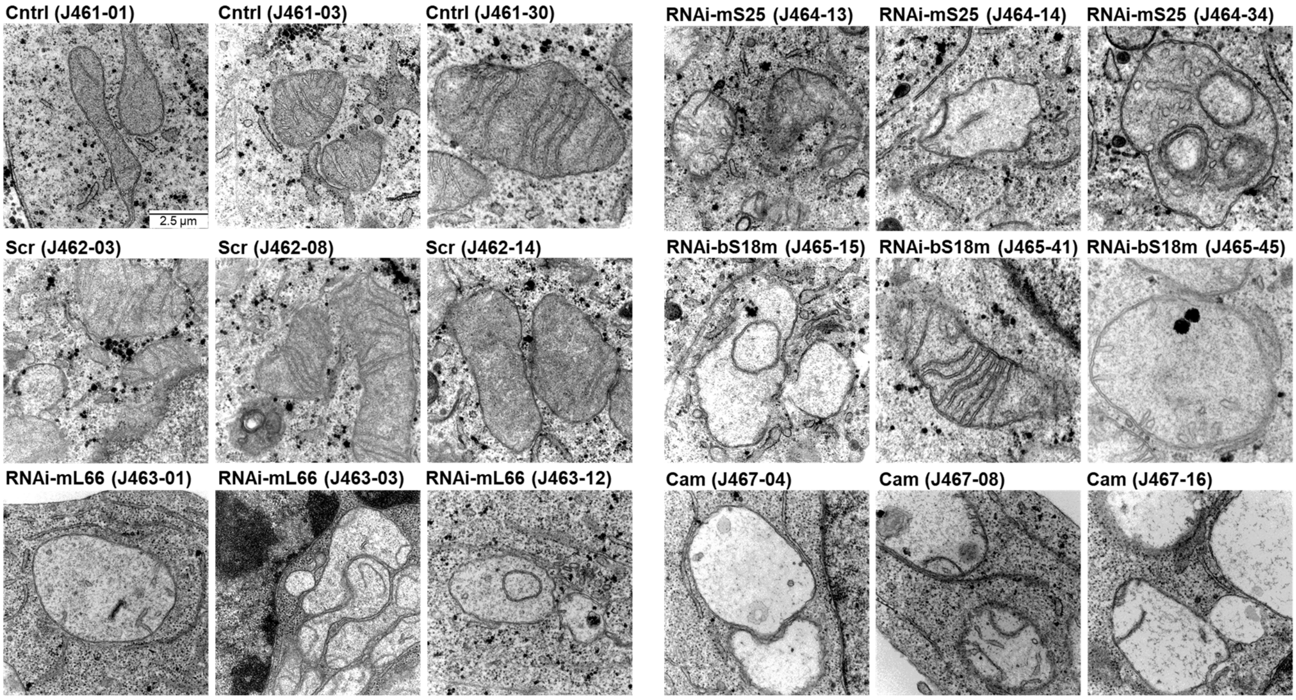

Figure S4. Mitochondria from HeLa cells depleted for Fe/S cluster-binding MRPs exhibit abnormal mitochondrial morphology. HeLa cells were depleted for mL66, mS25, or bS18m by two consecutive rounds of RNAi-mediated transfection as in Fig. 3 and analyzed by electron microscopy after a total of six days of cultivation. Control cells were transfected with scrambled siRNA (Scr) or were mock-transfected (Cntrl). As a reference for cells lacking mitochondrial protein translation, the specific inhibitor chloramphenicol (Cam) was added (cf. Ref. (43)). Sets of three representative images are shown. Mitochondrial morphology was substantially altered in MRP-depleted or CAM-treated cells.

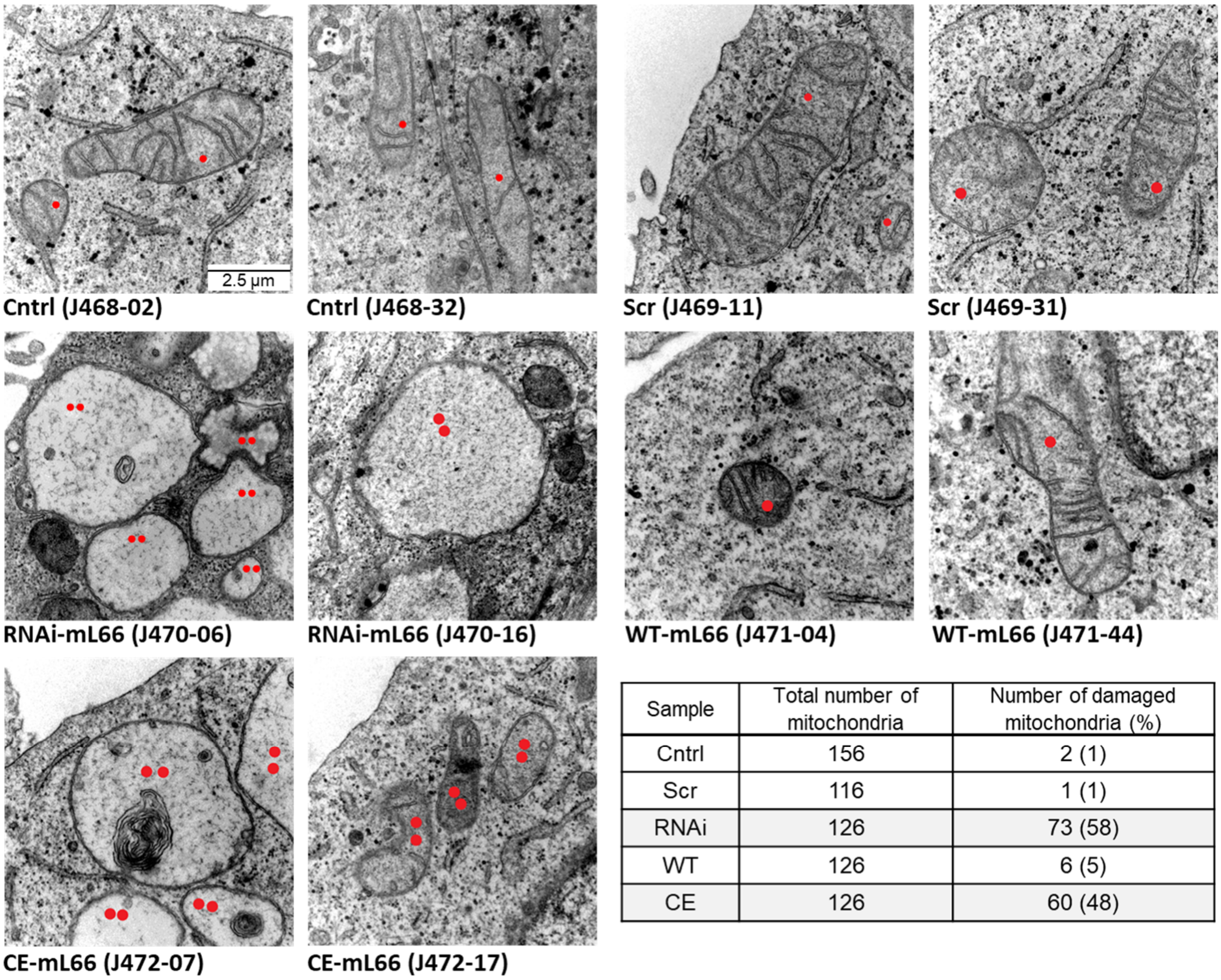

Sample	Total number of mitochondria	Number of damaged mitochondria (%)
Cntrl	156	2 (1)
Scr	116	1 (1)
RNAi	126	73 (58)
WT	126	6 (5)
CE	126	60 (48)

Figure S5. Quantitation of mitochondria with altered morphology in HeLa cells depleted for mL66. HeLa cells were mock-transfected (Cntrl), received scrambled siRNA (Scr), or were transfected with siRNA against mL66 either alone (RNAi) or in addition with complementing plasmids encoding wild-type mL66 (WT) or the Cys exchange mutant (CE) according to Fig. 3. After cultivation for six days with two rounds of transfection, cells were analyzed by electron microscopy, and 60 mitochondria-containing images were randomly captured per condition (two representative images per condition are shown). Mitochondria were counted and categorized as intact (one red dot) or damaged (two red dots). Mitochondria were counted as damaged, when they fulfilled at least two of the following three criteria: 1) less than three cristae related to size of cross section, 2) abnormal cristae inner membranes, cristae membranes with onion shape or other mitochondria-internal structures, and 3) weak staining of matrix space.

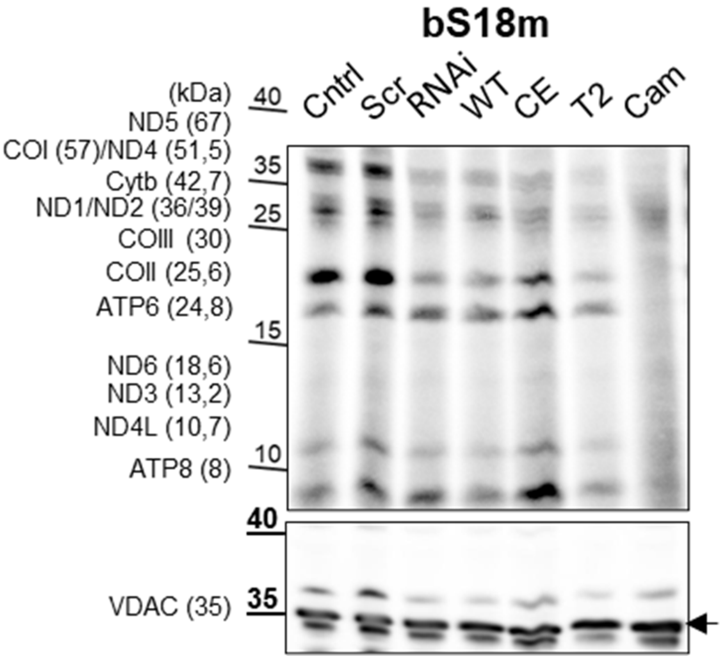

Figure S6. The mitoribosomal protein bS18m is required for mtDNA-dependent protein synthesis. Hela cells were mock-transfected (Cntrl), or treated with scrambled siRNA (Scr), a pool of siRNA against bS18m either alone (RNAi) or together with the respective complementing plasmids coding for wild-type bS18m protein (WT) or the Cys-exchange variant (CE) including the native 3'UTR, or a plasmid coding for bS18m transcript variant 2 (T2) which is a shorter variant, lacking the Cys residues. As a control, cells treated with the mitochondrial protein synthesis inhibitor chloramphenicol (Cam) were included. After ^{35}S-Met/Cys radiolabeling of proteins (see Fig. 5), an autoradiography of membrane-blotted whole cell lysates was obtained after SDS-PAGE. The lower part presents an immunostaining of VDAC1 as a loading control. Observed molecular masses (kDa) are indicated.

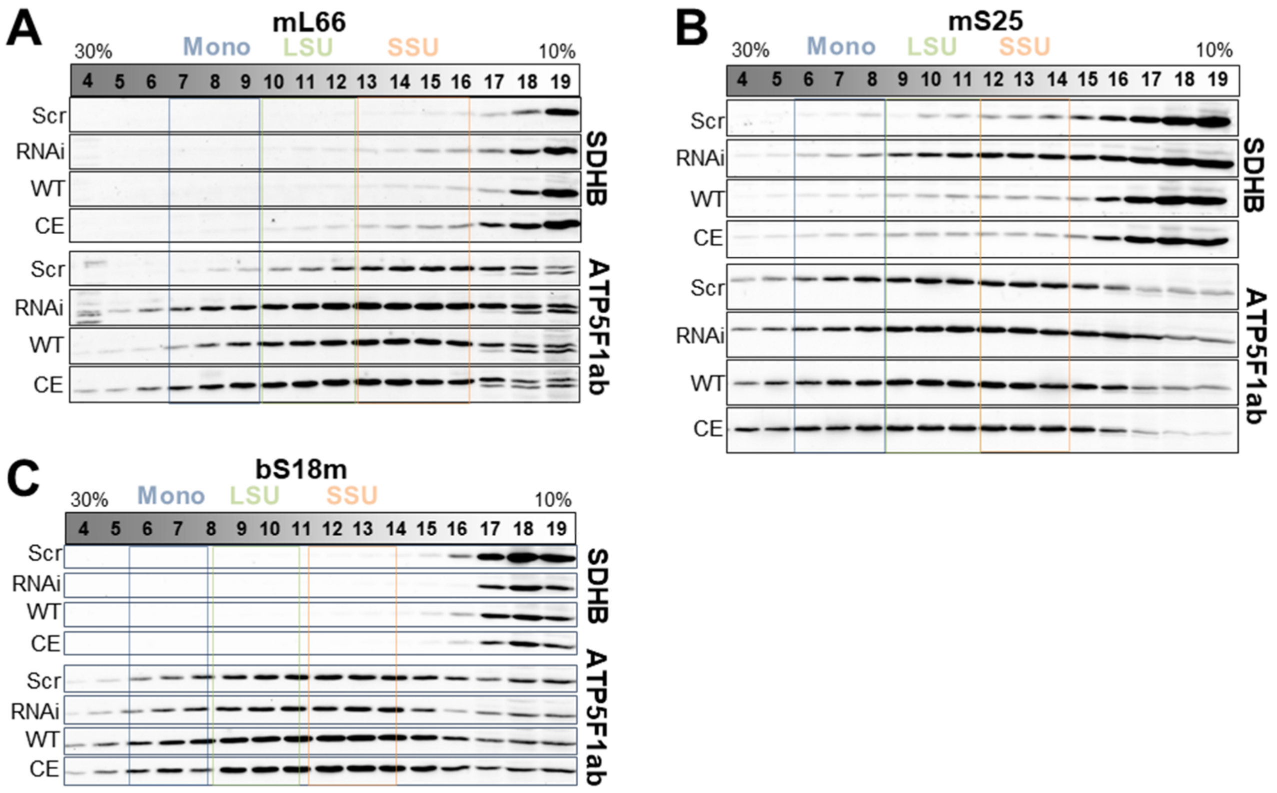

Figure S7. Immunostaining of non-mitoribosomal mitochondrial proteins from sucrose density gradient analyses of MRP-depleted and -complemented cells. Representative immunoblots from **A.** mL66, **B.** mS25, or **C.** bS18m depletion-complementation experiments after fractionation of mitochondrial extracts by sucrose density gradient centrifugation (see legends of Fig. 7 and Fig. S8 for sample preparation). The non-mitoribosomal α/β subunits of ATP-synthase ATP5F1AB (56/50 kDa) and respiratory complex II subunit SDHB (30 kDa) were immunostained to monitor the effects of the MRP depletion-complementation on other mitochondrial proteins. Consistent with the results of Fig. 4, SDHB levels were also decreased in case of bS18m depletion and complementation, for an unknown reason.

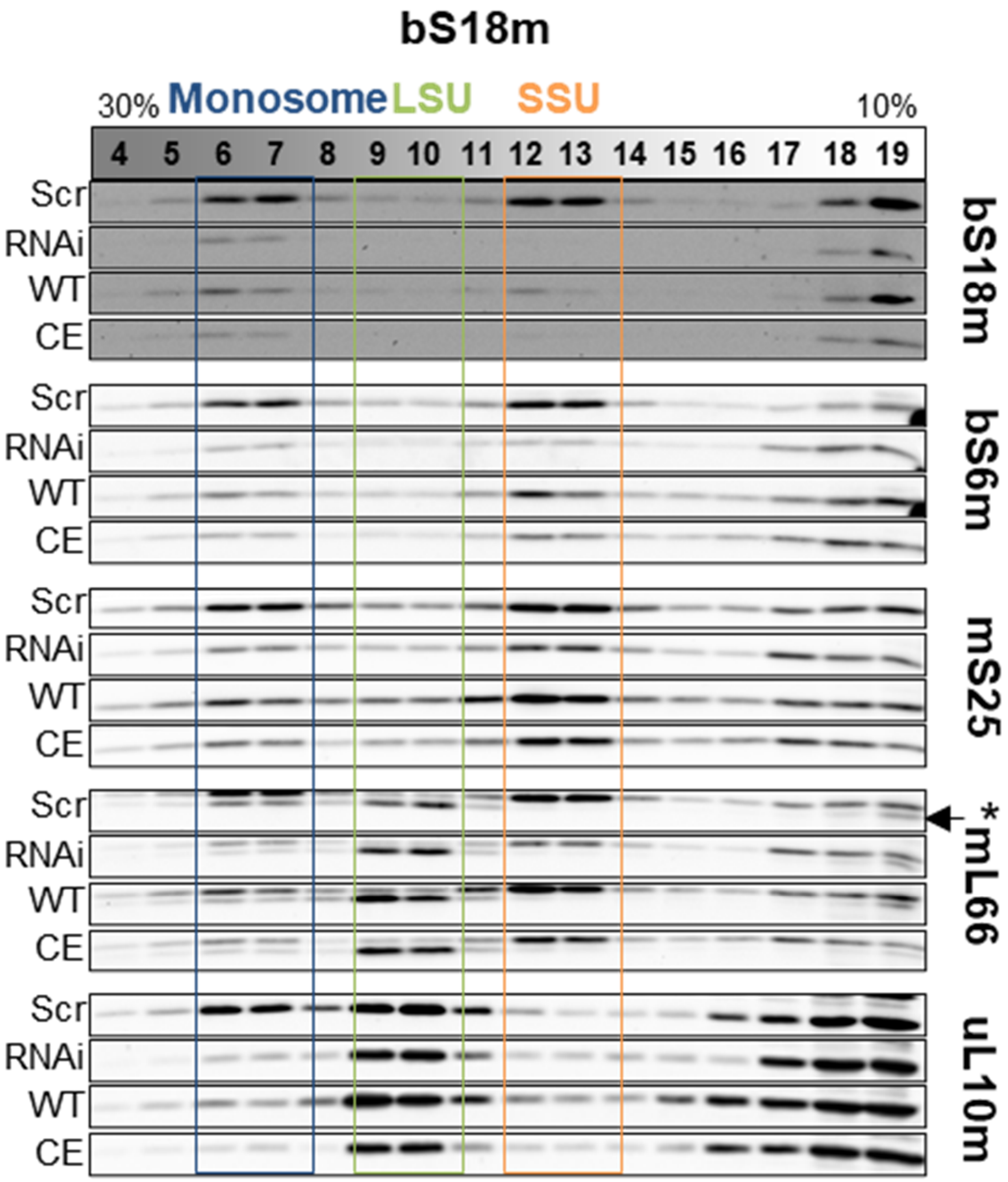

Figure S8. Testing the role of bS18m in mitoribosome assembly and/or stability. HeLa cells were treated with siRNA and complementing plasmids for the mitoribosomal subunit bS18m as described in Fig. 3, and mitoribosomal composition was analyzed by sucrose density gradient centrifugation followed by immunoblotting according to Fig. 7. Fractions with monosome, large and small ribosomal subunits (LSU and SSU, respectively) are indicated. Representative immunoblots are shown. Observed molecular masses: bS18m (15 kDa), bS6m (14 kDa), mS25 (19 kDa), mL66 (18 kDa) and uL10m (30 kDa). Scr, scrambled control siRNA; RNAi, siRNA-mediated depletion; WT, complementation of siRNA-mediated depletion by wild-type protein; CE, complementation of siRNA-mediated depletion by Cys-exchange variant. The specific signal for mL66 is marked by an arrow; the upper band in this blot originates from preceding mS25 staining.

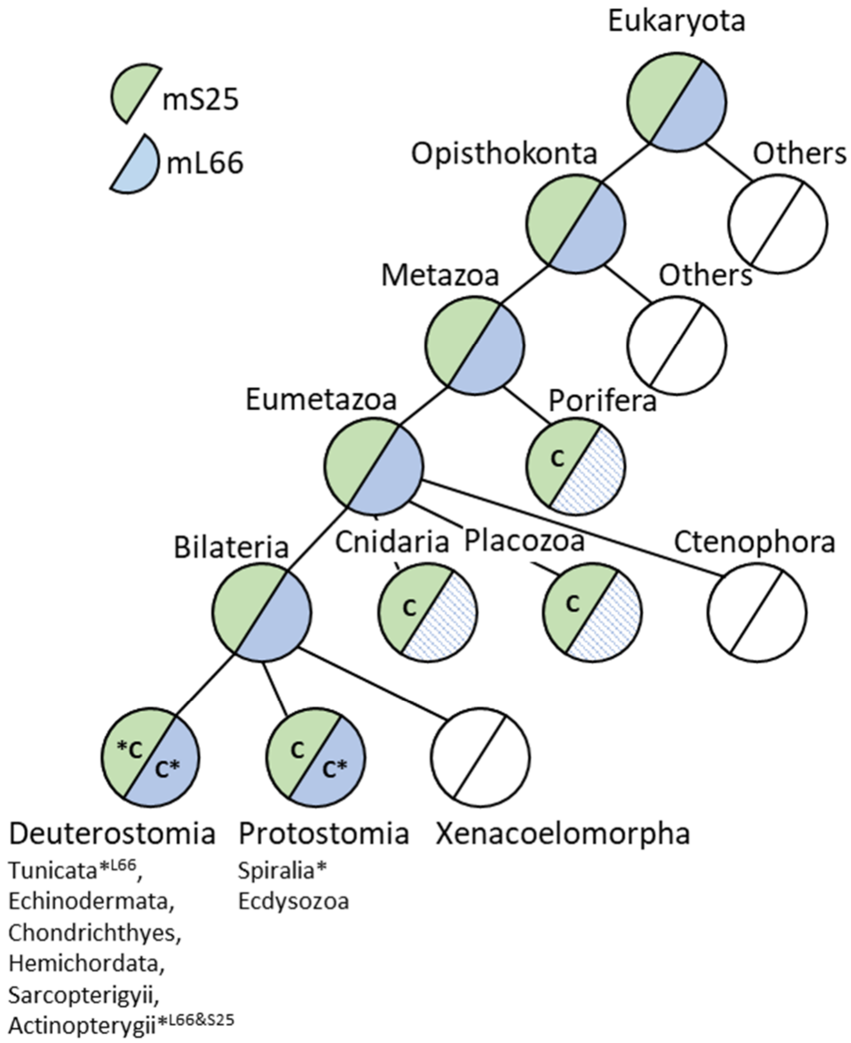

Figure S9. Overview of the phylogenetic distribution of mL66 and mS25 mitoribosomal proteins. Sequence relatives of mL66 and mS25 from distinct clades were retrieved using the NCBI ortholog or NCBI BLAST searches. Blue color indicates the presence of mL66, green denotes the presence of mS25, and white the absence of both proteins. For clades represented by blue-shaded half circles, only few representatives of mL66 were found, and clear distinction from bS18m or mS40 proteins was not always possible. **c** indicates the presence of the Cys motif in mL66 or mS25. Asterisks indicate that in few species the conserved Cys motif was missing, e.g. in Actinopterygii (further discussed in the text), and in case of mL66 also in Tunicata and Spiralia.

A mL66

```
Hsap  TTIIEGRITATPKES-------------PNPPNPSGQCPICRWNLKHHKYNYDDVLLLSQFIRPHGGMLPRKITGLCQEEHRKIEECVKMAH
Btau  TTIIEGRITGTPKES-------------PNPPNPSGQCPICRWNLKHHKYSYEDVLLLSQFIRPHGGMLPRSITGLCQEEHRKIEECVKMAH
Sscr  TTIIEGRITETPQES-------------PNPPNPTGQCPICRWNLKHHKYNYEDVLLLSQFIRPHGGMLPRRITGLCQEEHLKIEECVKMAH
Mmus  TTVIEGRITETPKAT-------------PDPPNPSGQCPICRWNLKHHKYTYEDVLLLSQFIRPYGGMLPRRVTGLCREEHRKIEECVKMAH
Ggal  TTIIEGKIIED-TET-------------PTPPNPSGQCPICRWNLKHHKYDYVDVLLLSQFIRSDGGMLPRRITGLCLEEHKKIAACVQMAH
Ecal  TTLIGGKIVED-KPT-------------HDPPNPSGQCPICRWNLKHHKYNYTDVLVLSQFIRPDGGMLPRRVTGLCSEEHKKIEVCVKMAQ
Frub  TTTIQGVILDI-PEA-------------PQPPNPVAKCPIYRWNLQHKYNYTDVLLLSQFIRSDGGMLPKRITGLCPEEHRKIAICVQMAH
Drer  TTIIEGFIETT-TEQ-------------PQPPNPTASCPIYRWNLQNKYNYTDVLLLSQFIRSDGGLLPRRITGLCAQEHNKIAICVQMAH
Ipun  TTVIEGLTVAA-PES-------------PQPPNPTAKCPIYRWNLQNKYTYTDVLLLSQFIRSDGGMLPRRITGLCAQEHRKIAICVQMAH
Sfru  SIVIEAVNVPSPRTELLVRAENLPNHEPAVSPSEKPPCYMCALGLDV--KHTDVLILSQFVRSDGCMLPRRITGLCRRQQKKMGKLVTMAQ
Dmel  VKIFEGVNVESPRAHLMLKSA-----------CQTKFCPECTLGLDI--KHTDVLILSQYVRSDGCMLPRRITGLCHRQQKKMGTLVTMAQ
Cele  TYISMEKVTDS-----------------RGARKDDELCSLCTCNVPIKLTYKDVLILEQFMRDDGTVLPRQLTGLCKKQQLRMERCVMQAF
                                           *     .:     : ***:*.*::*  * :**: :**** .:: ::   *  *
```

uL10m

```
Hsap  IPPK--PAIH--PSCLPSPPSP------PQEEIGLIRLLRREIAAVF--QDNRMIAVCQNVALSAEDKLLMRH  114
Btau  IAPK--PVVN--PRCLPPPPSP------PQEETGLIRLLRREIAAVF--RDNRMIAVCQNVAMSAEDKLLMRH  114
Sscr  IPPK--PTIN--PRCLPPPPTP------PQEETGLVRLLRREIAAVF--RDNRMIAVCQHVALSAEDKLLLRH  168
Mmus  IPPK--PAIN--PRCLPPPPKP------PKEESGLVRLLRQDIVAVF--RDNRMIAVCQNVALSAEDKLLLRH  114
Ggal  IAPR--PAVP--ERCLAPRRKV----EEEEEEYGYARLLRQQVEEAF--RDNRMIAVCQYNSMPGEDMVLMRH  111
Ecal  IPPK--PVIP--DRCLKPLPKR------NQEESGLVRILKREVDMMF--QNNKMIAVFQNNSSSSEDLLLIKY  125
Frub  IPPA--RPIP--PGVYSPRTED------CKEENPFLLFLKKEVQKVF--QECKMIAVVQNNACKSEDMIILKH  110
Drer  IPPT--LSAP--PAALAPRVKK------TGEESSLAVMLRKDLENLF--QEYKMIAVAQNNAISAEDMIHLKH  110
Ipun  IPPK--PAAP--PGAFTPRMQK------TKEESGLERLLKRDLESVF--SESKMIAVLQNNATNAEDMLLLKH  110
Sfru  KYGTPKYLLPDAALCDRG-EKK----YAREIDNPFERILANECLNWF--NTSKMVIFLHVNAISMEDKMPVFA  112
Dmel  KYPE----LPKAKSCFKTRAER----TQQQQENPYNEIIAREVRNWL--DHSRLVAFFHLSSITADDIFRVRV  112
Cele  ELPP------TVNMCIPPGLIKREHTGKSKEYSDVELALSNLVRDWMVREEFRVMAVCQFLPVPGRTLWFAKN  107
                                            : .     :     ::: . :
```

Figure S10. The Cys motif in mL66 and uL10m proteins is conserved in metazoans, except for modern ray-finned fish.

A. Multi-sequence alignments of mL66 and uL10m proteins from mammals: *Homo sapiens* (Hsap), *Bos taurus* (Btau), *Sus scrofa (*Sscr), *Mus musculus (*Mmus); bird: *Gallus gallus* (Ggal); fish: *Erpetoichthys calabaricus* (Ecal), *Fugu rubripes* (Frub), *Danio rerio* (Drer), *Ictalurus punctatus* (Ipun); insects: *Spodoptera Frugiperda* (Sfru), *Drosophila melanogaster* (Dmel); and worm: *Caenorhabditis elegans* (Cele). Conserved Cys residues coordinating the [2Fe-2S] cluster in the human mitoribosome are highlighted in grey. **B.** Comprehensive alignment of 217 mL66 proteins from vertebrates as circular tree representation, with symbols showing the presence of the Cys motif in mL66 and uL10m, the presence of mL66 paralogs, or when no uL10m sequence was found in the database, as indicated in the legend (left); created with iTOL (82). TCLA: Turtles, crocodiles, lizards and amphibians; CCL: Coelacanth, cartilaginous fish, lamprey. In many ray-finned fish (Actinopterygii), the second Cys of the mL66-motif is replaced by Tyr, and the fourth Cys ligand provided by uL10m is absent (conserved motif black and variant motif blue box and half-circle). Only some ancient species contain the full Cys motif (i.e. elephant shark, lamprey, reedfish), or a partial motif (i.e. coelacanth, spotted gar, *Asian bonytongue* and *Paramormyrops kingsleyae*).

Figure S10B: Legend see above.

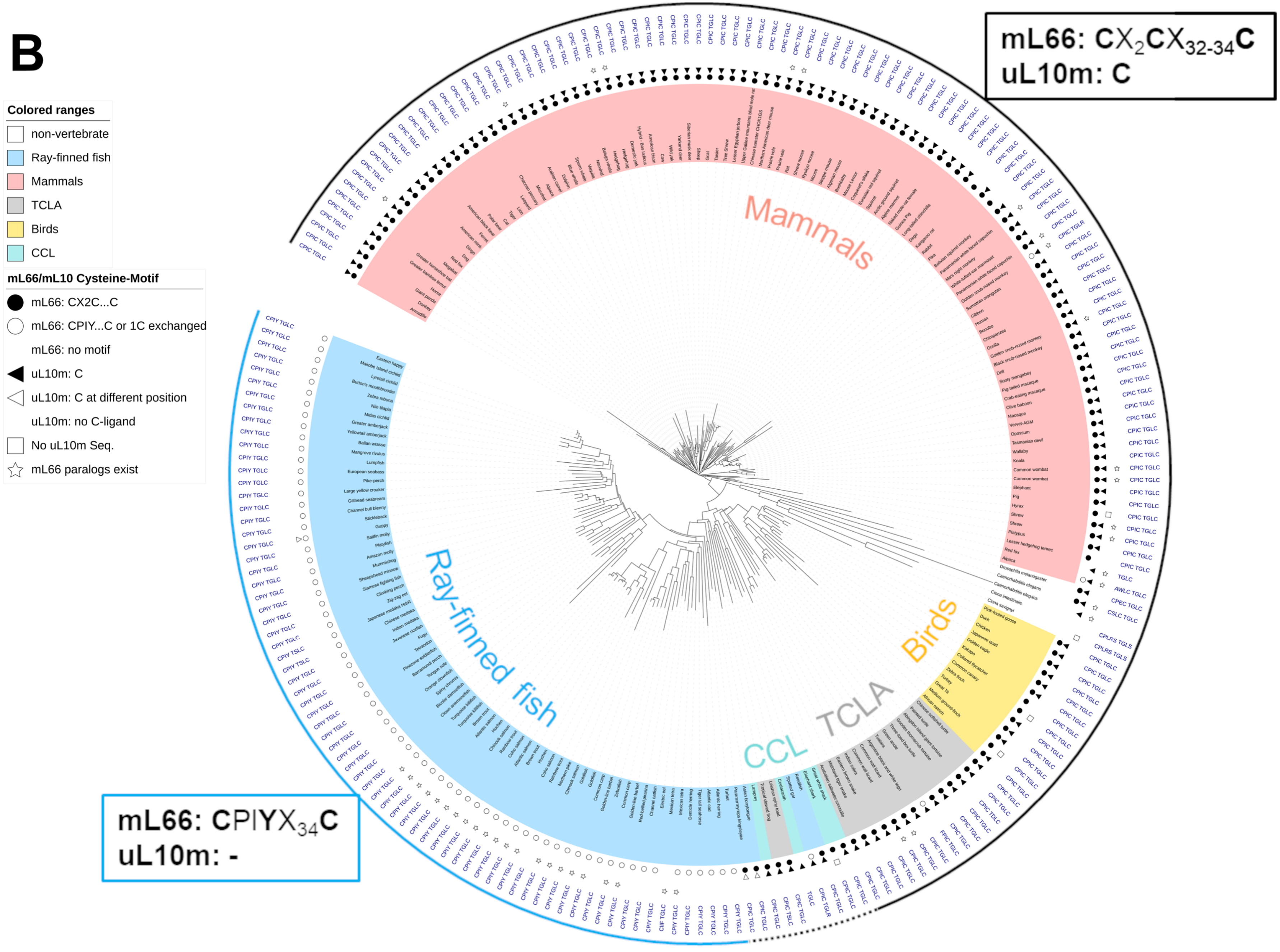

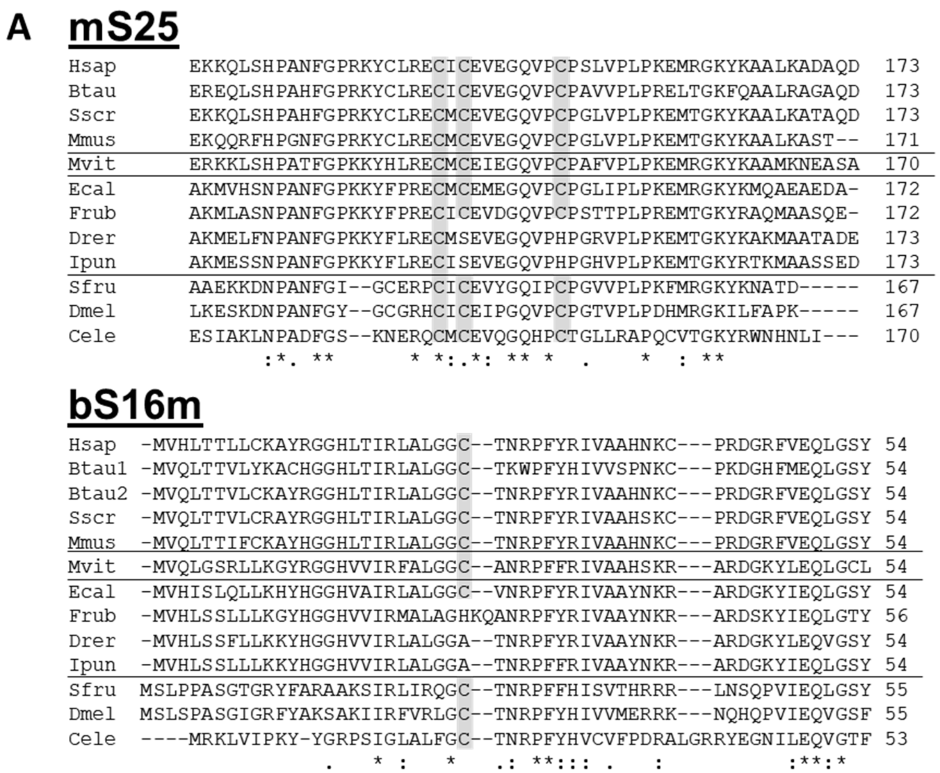

Figure S11. The Cys motif in mS25 and bS16m proteins is conserved in metazoans, except for modern ray-finned fish.

A. Multi-sequence alignments of mS25 and bS16m proteins from mammals: *Homo sapiens* (Hsap), *Bos taurus* (Btau), *Sus scrofa* (Sscr), *Mus musculus* (Mmus); bird: *Manacus vitellinus* (Mvit); fish: *Erpetoichthys calabaricus* (Ecal), *Fugu rubripes* (Frub), *Danio rerio* (Drer), *Ictalurus punctatus* (Ipun); insects: *Spodoptera Frugiperda* (Sfru), *Drosophila melanogaster* (Dmel); and worm: *Caenorhabditis elegans* (Cele). Conserved Cys residues coordinating the [2Fe-2S] cluster in the human mitoribosome are highlighted in grey. **B.** Comprehensive alignment of 206 mS25 proteins in vertebrates as circular tree representation, with symbols showing the presence of the Cys motif in mS25 and bS16m, the presence of mS25 paralogs or when no bS16m sequence was found in the database, as indicated in the legend (left); created with iTOL (82). TCLA: Turtles, crocodiles, lizards and amphibians; CCL: Coelacanth, cartilaginous fish, lamprey. In most ray-finned fish (Actinopterygii) the fourth Cys ligand provided by bS16m is replaced by His (conserved motif black and variant motif blue box and half circle), and in the sub clade of Otocephala two Cys of the conserved motif in mS25 and the one Cys ligand in bS16m are replaced by Ser, His, or Ala (violet box and circle). Only some ancient fish contain the full Cys motif like mammals (coelacanth, lamprey, elephant shark, spotted gar, reedfish, *Asian bonytongue*, and *Paramormyrops kingsleyae*).

Figure S11B: Legend see above.

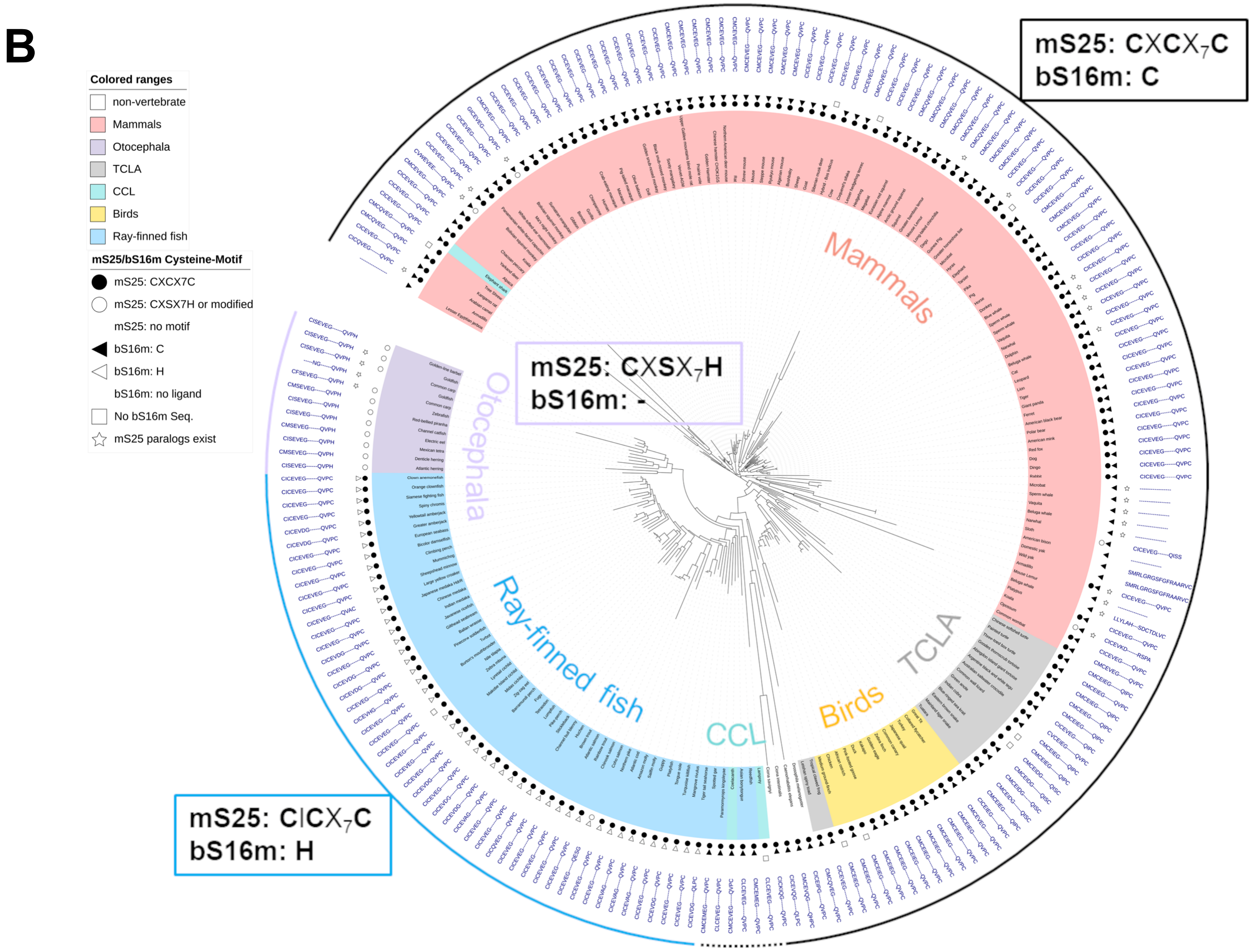

A **bS18m**

```
Hsap  41  ---SQQVSSNEDLPISMENPYKEPLKKCILCGK---HVDYK-NVQLLSQFVSPFTGCIYGRHITGLCGKKQKEITKAIKRAQIMGFMPVTYKDPAYLKDPKVCNIRYRE*- 142
Btau  41  --QCKQVTSSEDLPIPMENPYKEPLKKCILCEK---RVDYK-NVQLLSQFISPFTGCIYGRHITGLCGKKQREITKAIKRAQILGFMPVTYKDPAYLKDPKVCNIRYRE*- 143
Sscr  41  --QYKQVASNEDLPVPMENPYKEPLKKCILCEK---HVDYK-NVQLLSQFISPFTGCIYGRHITGLCGKKQKEITKAIKRAQILGFMPVTYKDPAYLKDPKVCNIKYRE*- 143
Mmus  41  --QFEQVTSNEDLPVPMENPYKEPLKKCVLCEK---RVDYK-NVQLLSQFISPFTGCIYGRHITGLCGKKQREITKAIKRAQKMGFMPVTYKDPAYLKDPRVCNIRYRE*- 143
Ggal  46  QQAA----AESDLPIEMENPYEEPPKRCILCGI---NVDYK-NVQLLSQFVSPYTGRIYGRHITGLCNKKQKEISKAIKRAHILGFMPVMFKNPSFLTDPKICNVKY*--- 144
Mcan  30  EGQPQAQPEHPDQPIQMENPYKEPPKRCILCKI---NVDYK-NVQLLSQFVSPYTGCIYGRHITGLCHKKQREVTKAIRRAQVLGFMPVVLKNPQFLTDPKICNIKYPE*- 134
Pmaj  30  EGQP----ERSDQPIHMENPYKDPPKRCVLCGI---NVDYK-NVQLLSQFVSPYTGSIYGRHITGLCNKKQKEITKAIKRAHVFGFMPIMFKNPQFLTDPKLCNIKHPE*- 130
Frub  26  TVKPDVAKEDDGVLVKMENPYKEPQKGCLLCNV---TVDYK-NIQLLSQFISPHTGHIYARHFTGLCGVKQKQVAKAIKKAQSMGFMSVTHKHPQFMRDPNICAVKQLN*- 130
Drer  32  TTSQQDPASKDDMPIKMENPYKQPAKTCILCNV---TVDFK-NVQLLSQFISPHTGRIYGRHITGLCGRKQKEVTKAIKKSRSMGFMSVTLKDPLFIQDPDICGTKHGE*- 136
Ipun  32  SSTIQE-VKLNDMPIQMENPYKQPQKGCILCNV---SVDYK-NVQLLSQFISPHTGRIYGRHLTGLCGRKQREVTKAIKRARSMGFMSVTLKDPQFIKDPNVCDIRHME*- 135
Sfru  26  --TDTKLSSNDDEPVAMPNPYKKERRQCILCKLNI-KPDYK-NYRLLSQFQSPYTGRIYGRHITGLCKTKQEQVESEIKKAQNCAYMPYYYKDKTFLKDPKLFDPENPIRS 132
Dmel  31  --SAAPESSDLDLPIDIKNPYEKDPQQCILCKHSI-EPHYK-NVKLLSQFQSPYTGRIYGRHITGLCKRRQEQVEQAILRAQQCLLMPGYHKDLDFLQDPKLFDPERPVRP 137
Cele  27  TQGSPSVSSDDEPVILENNPYTKEPRKSFLCSTGV-ELDYK-NSRLLQQFVSTFSGRVYDRHITGLCDENKKKLIEAIAKSRRAGFMPIFVKDPKYTRDPKLFDPLKPIRP 135
Scer  53  TIYDPFDFSMGRIHLDRKYQANKNSNRNDIMKSGANPLEFYARPRILSRYVTS-TGRIQHRDITGLSAKNQRRLSKAIRRCQAIGLM*---------------------- 138
Ecol   0  ---------------MARYFR--RRKFCRFTAEGVQEIDYK-DIATLKNYITE-SGKIVPSRITGTRAKYQRQLARAIKRARYLSLLPYTDRHQ*---------------- 75
Mpne  16  TTETTFNREEGKRMVRPLFKR--SKKYCRFCAIGQLRIDLIDDLEALKRFLSP-YAKINPRRITGNCQMHQRHVAKALKRARYLALVPFVKD*------------------ 104
Mtu1   0  ------MAKSSKRRPAPEKPV--KTRKCVFCAKKDQAIDYK-DTALLRTYISE-RGKIRARRVTGNCVQHQRDIALAVKNAREVALLPFTSSVR*---------------- 84
Mtu2   0  ------MA----AKSARKGPT--KAKKNLLDSLGVESVDYK-DTATLRVFISD-RGKIRSRGVTGLTVQQQRQVAQAIKNAREMALLPYPGQDRQR–RAALCP*------- 88
                                      .   :        .       *  : :   . :    .**      :. :   :  ..:    :
```

bS6m

```
Hsap  72  TAAVESMVEHLSRDIDVIRGNIVKHPL-TQELKECEGIVPVPLAEKLYSTKKRKK-----       125
Btau  94  TTTVASIMEHLSRDIDVIRPNVVKHPL-TQEVKECEGIVPVPLEEKLYSTKKRK------       146
Sscr  71  ATTVESMMEHLSRDIDVIRPNIVKHPL-TQEVKECEGIVPVPLEEKLYSTKKRK------       123
Mmus  72  TSAVENILEHLARDIDVVRPNIVKHPL-TQEVKECDGIVPVPLEEKLYSTKRRKK-----       125
Ggal 231  PSIVSTMMDHLGRDIDIIRRAFIKYPV-SK-TEECSGIVPVNCEDKLIPKKK--------       280
Mcan  72  PSIVSAMTEHLGRDIDVIRQGFVKHPL-AK-PEECPGMIPVSYEDKLSGRKK--------       121
Pmaj  97  PSIVSPMMDHLGRDIDVIRRAFIKHPL-AK-AEECGGITPVSPEEKLSSKKN--------       146
Frup  72  PNMITGLLNHLHLDIDVVRPTVLKTDE-QVSKKNCCGLQQ--------------------       110
Drer  72  PNIVSGLLNHLERDVDVLRPTVVKKDT-ELPKGQCCGVAREVKAKMAS------------       118
Ipun  72  PSILTALLNHLERDVDVVRQTVLKKDS-ELPKGPCCASLPSQTN----------------       114
Sfru  72  TKAVSDLKEEYSRDVDVIRQRVFKCEE-SS-SNNCT------LEEELLPPAYRDEVKKMI       123
Dmel  72  PTKIADLKEEFGRDIDIIRRYIFKVEE-PE-QKPCT------LHEEMLPPAYRKDVQEII       123
Cele  74  REARQKTKSILTHDLDTVQVDLIQVDTLPAPKVECN------LEEILKSPAERQAVKDLR       127
Scer  75  AAVQSEILRTLKKDPRVIRSSIVKVDL-DKQLDRASSLH-----RSL----GKKSILELV       124
Ecol  68  QEVIDELETTFRFNDAVIRSMVMRTKH---AVTEASPMVKAKDER-R---ERRDDFANET       120
Mpne  66  NQSTKDFKRTANINKQVLRELIINLER---EYGYLASINPKKQQLAL---QKRAKYDEII       119
Mtu   69  PATVSELDRQLSLNESVLRTKVMRTDK---H-----------------------------       96
                     :   ::  ...
```

Figure S12. The Cys motif in bS18m-bS6m proteins is conserved in metazoans.

A. Multi-sequence alignments of bS18m and bS6m proteins from mammals: *Homo sapiens* (Hsap), *Bos taurus* (Btau), *Sus scrofa* (Sscr), *Mus musculus* (Mmus); birds: *Gallus gallus* (Ggal), *Manacus candei* (Mcan), *Parus major* (Pmaj); fish: *Fugu rubripes* (Frub), *Danio rerio* (Drer), *Ictalurus punctatus* (Ipun); insects: *Spodoptera frugiperda* (Sfru), *Drosophila melanogaster* (Dmel); worm: *Caenorhabditis elegans* (Cele), and relatives from yeast: *Saccharomyces cerevisiae* (Scer) or bacteria: *rpsR* and *rpsF* from *Escherichia coli* (Ecol), *Mycoplasma pneumoniae* (Mpne) and *Mycobacterium tuberculosis* (Mtu, with RpsR paralogs Mtu1, Mtu2). Conserved Cys residues coordinating the [2Fe-2S] cluster in the human mitoribosome are highlighted in grey. **B.** Comprehensive alignment of 165 bS18m proteins from vertebrates and a few non-vertebrates as circular tree representation, with symbols showing the presence of the Cys motif in bS18m and bS6m and the presence of bS18m paralogs as indicated in the legend (left); created with iTOL (82). Most species contain multiple bS18m paralogs, some lacking the Cys motif, yet for better presentation only one paralog containing the motif was chosen. TCLA: Turtles, crocodiles, lizards and amphibians; CCL: Coelacanth, cartilaginous fish, lamprey. The majority of vertebrates (except for platypus), *D. melanogaster* and *C. elegans* contain the conserved Cys residues in bS18m and a fourth Cys ligand in bS6m.

Figure S12B: Legend see above.

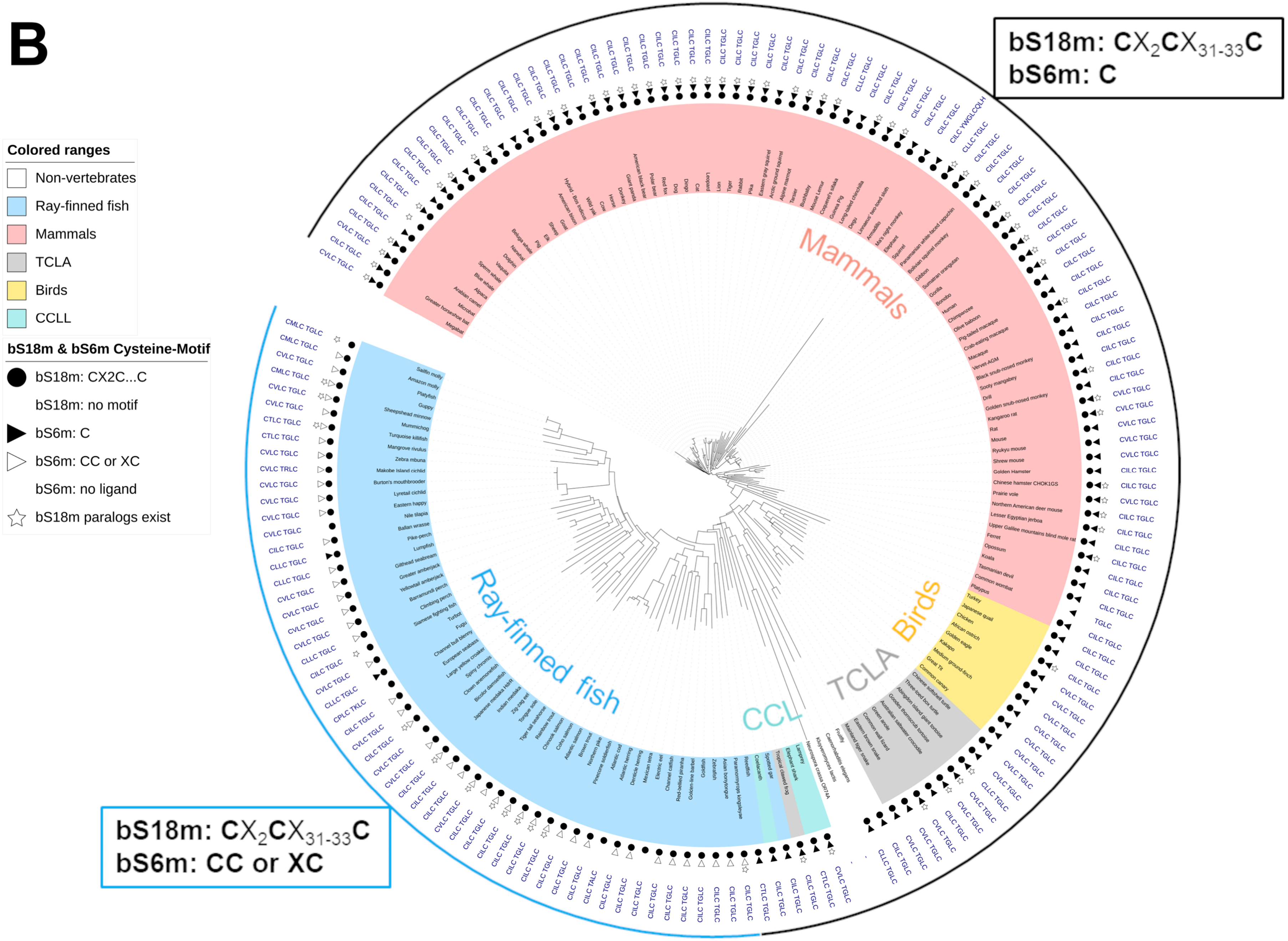

Supporting Tables

Table S1. Number of S18 ribosomal protein-related sequences with complete ($CX_2C…CX_2H$), altered or missing Cys motif.

Phylum/Supergroup	Total number of sequences	conserved Cys-motif	altered Cys-motif	without Cys-motif
Terrabacteria*	375	188	32	155
Campylobacterota	15	10	5	0
PVC*	29	11	7	11
Myxococcota	15	14	0	1
Spirochaetota	10	6	1	3
Nitrospirota	4	3	0	1
Thermotogota	9	9	0	0
Aquificota	9	9	0	0
Acidobacteriota	8	6	2	0
Thermodesulfobacteriota	26	26	0	0
Elusimicrobiota	2	2	0	0
Synergistota	8	8	0	0
Coprothermobacterota	1	1	0	0
CC*	1	1	0	0
Chrysiogenota	1	1	0	0
Deferribacterota	4	4	0	0
Dictyoglomota	1	1	0	0
Calditrichota	1	1	0	0
SAR*	11	1	0	10
Bdellovibrionota	3	0	2	1
Pseudomonadota	319	2	303	14
FCB*	97	1	86	10
Fusobacteriota	5	0	0	5
Cryptophyceae	1	0	0	1
Rhodophyta	1	0	0	1
Viridiplantae	6	0	0	6
Opisthokonta	48	21	0	27
NC**	368	13	256	99

The number of the predominant sequence motif is highlighted in grey. In unclear cases, the two most abundant motifs are highlighted. The asterisks (*) indicate the following supergroups: Terrabacteria; PVC: Planctomycetota, Verrucomicrobiota, Chlamydiota; CC: Caldisericota, Cryosericota; SAR: Stramenopiles, Alveolata, Rhizaria; FCB: Fibrobacterota, Cloacimonadota, Bacteriodota. NC**: bacterial species not yet classified. Sequences of prokaryotic S18 ribosomal proteins were gathered from the microbial genome database (80). Multi-sequence alignments of proteins were done with Clustal Omega (81), and sequences were manually searched for the presence of conserved Cys motifs.

Table S2. Small interfering RNA (siRNA) used in this study.

siRNA	Target sequence
MRPS25 (s34758)[1]<br>MRPS25 (s34759)[1]<br>MRPS25 (s34760)[1]	CTTCCTGCGATTCTACTTA<br>AGGTCATGACAGTGAATTA<br>GCAATAAGGAGATCATGGA
MRPS18A (s30334)[1]<br>MRPS18A (s30335)[1]<br>MRPS18A (s30336)[1]	GAGCAGGTCTATTACCAAA<br>GTATAACTATGACGATGTT<br>CAACTCAACCGGTACCTGA
MRPS18C (J-013131-09)[2]<br>MRPS18C (J-013131-12)[2]	CTTACACATCCCGGGACT<br>ATTTATGGAAGGCACATTA
MRPS18C (s27262)[1]<br>MRPS18C (s27263)[1]<br>MRPS18C (s27264)[1]	GCATGTAGATTATAAGAAT<br>GAAAGCATGTAGATTATAA<br>GAACCTCTTAAGAAATGTA

[1] Silencer® Select pre-designed small interfering RNA, purchased from ThermoFisher Scientific (Waltham, MA, USA).
[2] Dharmacon™ ON-TARGET plus siRNA, purchased from Horizon Discovery LTD (Cambridge, UK).

Table S3. Plasmid constructs used in this study.

Plasmid	ORF	Backbone	Use	Source/Reference
pVA-I	none	pHindB	Cell recovery after electroporation	(65)
pCMV3_bS18m	bS18m	pCMV3	Expression Vector	This work
pCMV3_mS25<br>pCMV3_mL66<br>pCMV3_bS18mt2	mS25<br>mL66<br>bS18mt2	pCMV3	Expression Vector	Sino Biological Inc., Beijing, China
pCMV6_bS18m	bS18m-FLAG	pCMV6	Expression Vector	OriGene Technologies Inc. (Rockville, Maryland, USA)
Su9-DSRed2	Su9-DSRed2	pDsRed2-C1	Expression Vector	This work.
pcDNA5_Su9-FLAG-EGFP-PEST	Su9-FLAG-EGFP-PEST	pcDNA5	Expression Vector	(33)
pcDNA5_MRPS27-FLAG	MRPS27-FLAG	pcDNA5	Expression Vector	(35)
pCMV3_bS18m3'UTR	bS18m-3'UTR*	pCMV3	Expression Vector	This work

* bS18m-3'UTR comprises nucleotides 21-1496 of the transcript variant 1 (NM_016067.4), including the coding region at nt 21-449.

Table S4. Primers for PCR-assisted mutagenesis of MRP cDNA sequences.

Purpose	Sequence 5'-3'
Cys → Ser in mS25	FPt: tcc ATC tct GAA GTG GAA GGG CAG GTG CCC agc <u>CCC AGC CTG GTG CCA TTA CCC</u><br>RPt: gct GGG CAC CTG CCC TTC CAC TTC aga GAT gga <u>CTC CCG CAG GCA GTA CTT TCG</u>
Cys → Ala in mL66	FP: gcc CCC ATC gcc CGT TGG AAC CTG AAG CAC AAG<br>RP: CTG GCC AGA GGG GTT AGG AGG
Cys→ Ala in bS18m	FP: TTG gct GGA AAG CAT GTA GAT TAT AAG AAT GTA<br>RP: GAT ggc TTT CTT AAG AGG TTC TTT ATA AGG ATT TTC
silent mutation (s34758) mS25	FPt: **T** CT**C** **A**G**G** TT**T** TA**T** **C**TT <u>GAT TCT GGG GAG CAG GTC CTG G</u><br>RPt: **A**A**G** **A**TA **A**AA **C**C**T** **G**AG **A** <u>AAG GGT GAC GGC GTC ATG TTC T</u>
silent mutation (s34759) mS25	FPt: **A** GT**T** ATG AC**T** GT**C** AA**C** <u>TAC AAC ACG CAT GGG GAG CTG G</u><br>RPt: **G**TT **G**AC **A**GT CAT **A**AC **T**<u>TT C ACG GAG TCC TTG AAC ACC ACG</u>
silent mutation (s34760) mS25	FPt: **TCT** AA**C** AA**A** GA**A** AT**T** <u>ATG GAG CAC ATC AGA AAA ATC TTG</u><br>RPt: **A**AT **T**TC **T**TT **G**TT **AGA** <u>CTT GGT CTC CAC ATC CAC CAG</u>
silent mutation (s30334) mL66	FPt: **C** GC**T** GG**A** **T**T**G** **C**T**T** CC**T** <u>AAT CAC AGG CCT CGG CTT CCT</u><br>RPt: **A**GG **A**A**G** **C**A**A** **T**CC **A**GC **G** <u>CGG TGG GCC ATC TTC ACA CAC T</u>
silent mutation (s30335) mL66	FPt: **A** TA**C** AA**T** TA**C** GA**T** GA**C** GT**C** <u>CTG CTG CTT AGC CAG TTC AT</u><br>RPt: **G**AC **G**TC **A**TC **G**TA **A**TT **G**TA **T** <u>TTG TGC TTC AGG TTC CAA CGG</u>
silent mutation (s30336) mL66	FPt: **G** CT**T** AA**T** **A**G**A** TA**T** CT**C** <u>ACG CGC TGG GCT CCT GG</u><br>RPt: **G**AG **A**TA **T**C**T** **A**TT **A**AG **C**<u>TG G GGT TTG CTC TTC GGA ACA ACT</u>
silent mutation (J-013131-09) bS18m	FPt: **C**AC **T**CA **C**CC **T**GG **A**AC **C**<u>CA CAC GGT GCT TTG GAG AAG AGG</u><br>RPt: **G**GT **T**CC **A**GG **G**TG **A**GT **G**<u>AG GCT GAC AGC AGC CGT TAC C</u>
silent mutation (J-013131-12) bS18m	FPt: **C** TA**C** GG**C** **C**G**C** CA**T** AT**C** <u>ACA GGT CTT TGT GGG AAG AAA CAG</u><br>RPt: **G** AT**A** TG**G** C**GG** CC**G** TA**G** <u>ATG CAT CCA GTA AAT GGA GAA ACA AAC</u>

Lower case letters: exchanged nucleotides for amino acid substitutions; bold letters: exchanged nucleotides for silent mutations; underlined sequence: part of tall primer that was used as respective small primer for SLIM PCR (69).

Table S5. Primary antibodies used in this study.

Antibody/antiserum<br>(host, dilution, order number)	Source	Validation
bS6m (rp, 1:750, 31764),<br>bS18m (rp, 1:600, 42908)	SAB® signal way antibody<br>(Greenbelt, MD, USA)	In house and this study<br>In house and this study
mL66 (rp, 1:750, 16235-1-AP),<br>mS25 (rp, 1:1,000, 15277-1-AP),<br>uL10m (rp, 1:750, 16652-1-AP),<br>VDAC1 (rp, 1:1,500, 55259-1-AP),<br>ATP8 (rp, 1:1,000, 26723-1-AP)	Proteintech Group<br>(Rosemont, IL, USA)	This study<br>This study<br>This study<br>(83)<br>Manufacturer and this study
NDUFS1 (mm, 1:500, sc-271510),<br>SDHB (mm, 1:1,000-2,500, sc-271548),<br>beta actin (mm, 1:750, sc-47778)	Santa Cruz biotechnology<br>(Dallas, TX, USA)	(33)<br>(33)<br>(68)
ACO2 (rp, 1:2,750, PA5-29037)	Invitrogen (ThermoFisher<br>Scientific, Waltham, MA, USA)	Manufacturer and<br>in house
NDUFB6 (mm, 1:1,750, ab110244)	Abcam (Cambridge, UK)	(84)
bS16m (rp, 1:500)	A. Saada	(57)
UQCR2 (rp, 1:2,500),<br>ATP5F1A/B A42 (rp, 1:1,250)	H. Schägger	(84)
MT-COX2 A85 (rp, 1:2,500),<br>ISCA2 (rp, ap, 1:50)	H. Schägger,<br>R. Lill	(27)
FXN (rp, 1:200)	R. Lill group	(31)
ISCU2 (rp, ap, 1:50)	R. Lill group	(85)

Abbreviations: rp: rabbit polyclonal, mm: mouse monoclonal, ap: affinity-purified.

Supporting References

27. Sheftel, A. D., Wilbrecht, C., Stehling, O., Niggemeyer, B., and Elsässer, H.-P., et al. Lill, R. (2012) The human mitochondrial ISCA1, ISCA2, and IBA57 proteins are required for 4Fe-4S protein maturation. *Molecular biology of the cell* **23**, 1157–1166
31. Stehling, O., Elsässer, H.-P., Brückel, B., Mühlenhoff, U., and Lill, R. (2004) Iron-sulfur protein maturation in human cells: evidence for a function of frataxin. *Human molecular genetics* **13**, 3007–3015
33. Schulz, V., Basu, S., Freibert, S.-A., Webert, H., and Boss, L., et al. Lill, R. (2023) Functional spectrum and specificity of mitochondrial ferredoxins FDX1 and FDX2. *Nature chemical biology* **19**, 206–217
35. Richter, R., Rorbach, J., Pajak, A., Smith, P. M., and Wessels, H. J., et al. Chrzanowska-Lightowlers, Z. M. (2010) A functional peptidyl-tRNA hydrolase, ICT1, has been recruited into the human mitochondrial ribosome. *The EMBO journal* **29**, 1116–1125
43. Storrie, B., and Attardi, G. (1973) Expression of the mitochondrial genome in HeLa cells: XV. Effect of inhibition of mitochondrial protein synthesis on mitochondrial formation. *The Journal of cell biology*, 819–831
57. Haque, E., Grasso, D., Miller, C., Spremulli, L. L., and Saada, A. (2008) The effect of mutated mitochondrial ribosomal proteins S16 and S22 on the assembly of the small and large ribosomal subunits in human mitochondria. *Mitochondrion* **8**, 254-261
65. Svensson, C., and Akusjärvi, G. (1985) Adenovirus VA RNAI mediates a translational stimulation which is not restricted to the viral mRNAs. *The EMBO journal* **4**, 957–964
68. Braymer, J. J., Stehling, O., Stümpfig, M., Rösser, R., and Spantgar, F., et al. Lill, R. (2024) Requirements for the Biogenesis of [2Fe-2S] Proteins in the Human and Yeast Cytosol. *bioRxiv*
69. Chiu, J., Tillett, D., Dawes, I. W., and March, P. E. (2008) Site-directed, Ligase-Independent Mutagenesis (SLIM) for highly efficient mutagenesis of plasmids greater than 8kb. *Journal of Microbiological Methods* **73**, 195–198
80. Uchiyama, I., Mihara, M., Nishide, H., Chiba, H., and Kato, M. (2019) MBGD update 2018: microbial genome database based on hierarchical orthology relations covering closely related and distantly related comparisons. *Nucleic acids research* **47**, D382-D389
81. Madeira, F., Pearce, M., Tivey, A. R. N., Basutkar, P., and Lee, J., et al. Lopez, R. (2022) Search and sequence analysis tools services from EMBL-EBI in 2022. *Nucleic acids research* **50**, W276-W279
82. Letunic, I., and Bork, P. (2021) Interactive Tree Of Life (iTOL) v5: an online tool for phylogenetic tree display and annotation. *Nucleic acids research* **49**, W293-W296
83. Torraco, A., Stehling, O., Stümpfig, C., Rösser, R., and Rasmo, D. de, et al. Carrozzo, R. (2018) ISCA1 mutation in a patient with infantile-onset leukodystrophy causes defects in mitochondrial 4Fe-4S proteins. *Human molecular genetics* **27**, 2739–2754
84. Sheftel, A. D., Stehling, O., Pierik, A. J., Netz, D. J. A., and Kerscher, S., et al. Lill, R. (2009) Human ind1, an iron-sulfur cluster assembly factor for respiratory complex I. *Molecular and cellular biology* **29**, 6059–6073
85. Navarro-Sastre, A., Tort, F., Stehling, O., Uzarska, M. A., and Arranz, J. A., et al. Lill, R. (2011) A fatal mitochondrial disease is associated with defective NFU1 function in the maturation of a subset of mitochondrial Fe-S proteins. *American journal of human genetics* **89**, 656–667
